# Urolithin A Hijacks ERK1/2‐ULK1 Cascade to Improve CD8^+^ T Cell Fitness for Antitumor Immunity

**DOI:** 10.1002/advs.202310065

**Published:** 2024-03-06

**Authors:** Shuaiya Ma, Qi Wu, Wenxian Wu, Ye Tian, Jie Zhang, Chaojia Chen, Xue Sheng, Fangcheng Zhao, Lu Ding, Taixia Wang, Laixi Zhao, Yuying Xie, Yongxiang Wang, Xuetian Yue, Zhuanchang Wu, Jian Wei, Kun Zhang, Xiaohong Liang, Lifen Gao, Hongyan Wang, Guihua Wang, Chunyang Li, Chunhong Ma

**Affiliations:** ^1^ Key Laboratory for Experimental Teratology of Ministry of Education and Department of Immunology, School of Basic Medical Sciences, Qilu Hospital, Cheeloo College of Medicine Shandong University Jinan Shandong 250012 P. R. China; ^2^ GI Cancer Research Institute Tongji Hospital Huazhong University of Science and Technology Wuhan Hubei 430074 P. R. China; ^3^ Guangdong Key Laboratory of Age‐Related Cardiac and Cerebral Disease Department of Neurology Affiliated Hospital of Guangdong Medical University Zhanjiang Guangdong 524001 P. R. China; ^4^ Shenzhen Research Institute of Shandong University Shenzhen 518057 P. R. China; ^5^ Advanced Medical Research Institute Cheeloo College of Medicine Shandong University Jinan Shandong 250012 P. R. China; ^6^ Central Laboratory Tongji University School of Medicine Tongji University Shanghai 200072 P. R. China; ^7^ Key Laboratory for Experimental Teratology of Ministry of Education and Department of Cell Biology School of Basic Medical Sciences Cheeloo College of Medicine Shandong University Jinan Shandong 250012 P. R. China; ^8^ Department of Physiology School of Basic Medical Sciences Shandong University Jinan 250012 P. R. China; ^9^ State Key Laboratory of Cell Biology Shanghai Institute of Biochemistry and Cell Biology Center for Excellence in Molecular Cell Science Chinese Academy of Sciences University of Chinese Academy of Sciences Shanghai 200031 P. R. China; ^10^ Key Laboratory for Experimental Teratology of Ministry of Education and Department of Histology and Embryology School of Basic Medical Sciences Cheeloo College of Medicine Shandong University Jinan Shandong 250012 P. R. China

**Keywords:** autophagy, CAR T cells, CD8^+^ cytotoxic T cells, ERK1/2, Urolithin A (UA)

## Abstract

According to the latest evidence, the microbial metabolite Urolithin A (UA), known for its role in promoting cellular health, modulates CD8^+^ T cell‐mediated antitumor activity. However, the direct target protein of UA and its underlying mechanism remains unclear. Here, this research identifies ERK1/2 as the specific target crucial for UA‐mediated CD8^+^ T cell activation. Even at low doses, UA markedly enhances the persistence and effector functions of primary CD8^+^ cytotoxic T lymphocytes (CTLs) and human chimeric antigen receptor (CAR) T cells both in vitro and in vivo. Mechanistically, UA interacts directly with ERK1/2 kinases, enhancing their activation and subsequently facilitating T cell activation by engaging ULK1. The UA‐ERK1/2‐ULK1 axis promotes autophagic flux in CD8^+^ CTLs, enhancing cellular metabolism and maintaining reactive oxygen species (ROS) levels, as evidenced by increased oxygen consumption and extracellular acidification rates. UA‐treated CD8^+^ CTLs also display elevated ATP levels and enhanced spare respiratory capacity. Overall, UA activates ERK1/2, inducing autophagy and metabolic adaptation, showcasing its potential in tumor immunotherapy and interventions for diseases involving ERKs.

## Graphical abstract

This study reports a new mechanism by which Urolithin A (UA) enhances the antitumor activity of primary CD8^+^ T and CAR T cells. UA directly binds and activates ERK1/2, further activating ULK1 to trigger autophagy and consequent metabolic adaption to maintain T cell fitness. These findings highlight the important role of UA‐ERK1/2‐ULK1 axis in tumor immunotherapy.

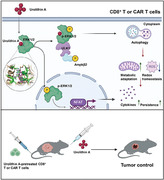

## 1 Introduction

Tumor immunotherapies employing immune checkpoint blockade (ICB) or adoptive cell transfer have shown significant clinical success across various malignancies, yet low response rates persist.^[^ ^1^ ^]^ It is well‐established that tumor immunotherapy aims to generate long‐term, potent CD8^+^ cytotoxic T lymphocytes (CTLs) closely linked to clinical tumor response.^[^ ^2^ ^]^

Recent studies have emphasized the pivotal role of the intestinal microbiota in modulating host immunity and influencing tumor immunotherapy efficacy.^[^ ^3^, ^4^, ^5^, ^6^, ^7^ ^]^ It has been shown that antibiotic treatment diminishes responses to anti‐PD‐1 immunotherapy,^[^ ^3^ ^]^ while certain bacterial strains elicit CD8^+^ T cell‐mediated antitumor response.^[^ ^8^ ^]^ The significance of microbial metabolites in enhancing antitumor immunity has been established. For instance, inosine, produced by intestinal *B. pseudolongum*, enhances the efficacy of ICB,^[^ ^9^ ^]^ and microbiota‐derived short‐chain fatty acid butyrate improves the memory potential of CD8^+^ T cells, enhancing adoptive immunotherapy for cancer.^[^ ^10^, ^11^, ^12^ ^]^ Additionally, the microbial metabolite trimethylamine N‐oxide was found to enhance CD8^+^ T cell‐mediated antitumor response in triple‐negative breast cancer by inducing pyroptosis of tumor cells.^[^ ^13^ ^]^ These findings underscore the potential of microbial metabolites as a therapeutic strategy to boost tumor immunotherapy, emphasizing the importance of the identification of potent bacterial metabolites regulating key TCR signaling pathways to augment antitumor responses.

Urolithin A (UA), a natural metabolite derived from human gut microbiota processing polyphenols in dietary products like pomegranate, berries, and nuts,^[^ ^14^ ^]^ was identified as a potent microbial metabolite in our investigation. UA administration has previously been linked to mitophagy induction, extending lifespan in *C. elegans* ^[^ ^15^ ^]^ and enhancing rodent muscle function.^[^ ^16^ ^]^ Preclinical models have substantiated UA's efficacy in improving muscle, brain, and joint functions while mitigating inflammation.^[^ ^17^, ^18^ ^]^ Human clinical trials have established UA's favorable safety profile, bioavailability,^[^ ^19^ ^]^ and positive impact on mitochondrial and cellular health, as well as muscle strength.^[^ ^20^ ^]^ A recent study highlighted UA's ability to robustly promote the expansion of CD8^+^ T memory stem cells (T_SCM_) through Pink1‐dependent mitophagy.^[^ ^21^ ^]^ However, whether UA regulates T cell activation and effector function, along with the direct target protein and underlying molecular mechanism, remains unclear, warranting further research.

In the present study, we identify UA as a potent microbial metabolite that enhances CD8^+^ T cell effector function and survival, thereby intensifying antitumor activity. Mechanistically, UA directly binds with ERK1/2 kinases, enhancing their activation and subsequently phosphorylating and activating the autophagy initiator ULK1 to facilitate autophagy. Specifically, the UA‐ERK1/2‐ULK1 cascade reduces oxidative stress and enhances cellular metabolism, thereby improving the persistence and antitumor activity of CD8^+^ CTLs. These findings shed light on the molecular mechanisms underlying UA‐augmented antitumor activity in T cells, emphasizing the promising application of UA in antitumor immunotherapy.

## 2 Results

### 2.1 Urolithin A Directly Promotes T Cell Activation and Antitumor Activities of CD8^+^ CTLs

**Figure 1:**
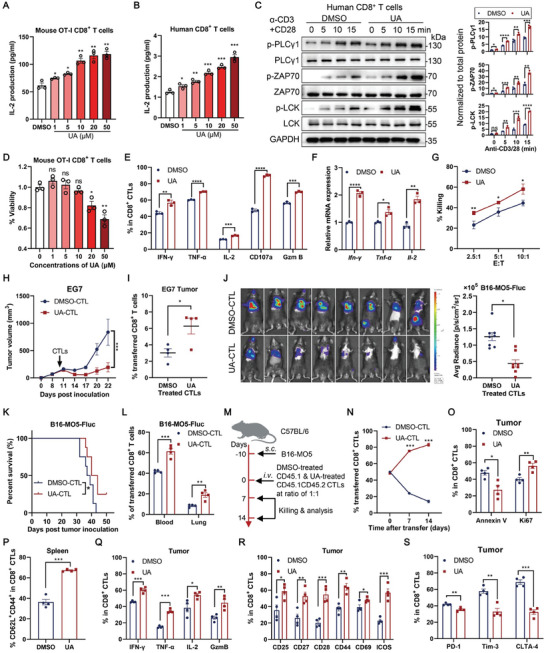
Urolithin A directly promotes T cell activation and antitumor activities of CD8^+^ CTLs. A) OT‐I CD8^+^ T cells were pretreated with DMSO or UA (1, 5, 10, 20, and 50 µm) for 48 h, followed by stimulation with 1 µg ml^−1^ anti‐CD3 and 3 µg ml^−1^ CD28 antibodies for 6 hours. IL‐2 production of OT‐I CD8^+^ T cells in supernatant was detected using ELISA. Data are presented as means ± SEM (*n* = 3) and were analyzed by two‐tailed unpaired Student's *t*‐test. B) Human CD8^+^ T cells were pretreated with DMSO or UA (1, 5, 10, 20, and 50 µm) for 48 h, followed by anti‐CD3/28 (1 + 3 µg ml^−1^) stimulation for 24 h. IL‐2 production of human CD8^+^ T cells in supernatant was detected using ELISA. Data are presented as means ± SEM (*n* = 3) and were analyzed by two‐tailed unpaired Student's *t*‐test. C) Representative immunoblot image (left) and quantification (right; normalized to total protein) of the indicated proteins in DMSO‐ and 10 µm UA‐treated human CD8^+^ T cells stimulated with anti‐CD3/28 antibodies (1 + 3 µg ml^−1^) for indicated time points. Data are presented as means ± SEM (*n* = 3) and were analyzed by two‐tailed unpaired Student's *t*‐test. All immunoblots were representative of three independent experiments. D) OT‐I CD8^+^ T cells were treated with DMSO or UA (1, 5, 10, 20, and 50 µm) for 48 h, followed by the evaluation of the cell viability. Data are presented as means ± SEM (*n* = 3) and were analyzed by two‐tailed unpaired Student's *t*‐test. E) stimulation for 6 h. The percentage of IFN‐γ, TNF‐α, IL‐2, CD107a, and Granzyme B (Gzm B)‐producing OT‐I CD8^+^ CTLs was assessed using flow cytometric analysis. Data are presented as means ± SEM (*n* = 3) and were analyzed by two‐tailed unpaired Student's *t*‐test. F) OT‐I CD8^+^ CTLs were treated with DMSO or 10 µm UA for 48 h, followed by anti‐CD3/28 stimulation for 6 h, and relative mRNA expression of *Ifn‐γ*, *Tnf‐α*, and *Il‐2* were detected using quantitative polymerase chain reaction. Data are presented as means ± SEM (*n* = 3) and were analyzed by two‐tailed unpaired Student's *t*‐test. G) OT‐I CD8^+^ CTLs were treated with DMSO or 10 µm UA for 48 h. Subsequently, cytotoxicity of DMSO‐ and UA‐treated OT‐I CD8^+^ CTLs against 10 nm OVA_257‐264_ peptide‐pulsed EL4 targets at indicated Effector: Target (E:T) ratios was detected in vitro. Data are presented as means ± SEM (*n* = 3) and were analyzed by two‐tailed unpaired Student's *t*‐test. H,I) OT‐I CD8^+^ T cells were treated with DMSO or 10 µm UA for 48 hours and adoptively transferred into C57BL/6 mice bearing subcutaneous EG7 tumor on day 10 post tumor inoculation (*n* = 4 mice per group). Tumor growth curves were monitored (H). The percentage of transferred CTLs in the tumor was assessed using flow cytometry (I). Data are presented as means ± SEM and were analyzed by two‐way analysis of variance (ANOVA) (H) and two‐tailed unpaired Student's *t*‐test (I). J–L) OT‐I CD8^+^ T cells were treated with DMSO or 10 µm UA for 48 h and adoptively transferred into B16‐MO5‐Fluc lung metastases‐bearing C57BL/6 mice on day 10 post tumor inoculation. Tumor growth indicated by luciferase activity in lung (J) and survival curve (K) were monitored (*n* = 8 mice per group). The percentage of transferred CTLs in peripheral blood and tumor was determined using flow cytometry (L) (*n* = 4 mice per group). Data are presented as means ± SEM and were analyzed by two‐tailed unpaired Student's *t*‐test (J,L) and Log‐rank test (K). M–S) Schematic diagram of co‐transfer of DMSO‐ (CD45.1) and 10 µm UA‐treated (CD45.1/2) OT‐I CTLs at ratio of 1:1 to subcutaneous B16‐MO5 tumor‐bearing C57BL/6 mice on day 10 post inoculation (M). The percentage of transferred DMSO‐ and UA‐treated CTLs in the tumor was monitored on days 7 and 14 (*n* = 4) (N). Percentages of Annexin V^+^ and Ki67^+^ transferred CD8^+^ CTLs in tumors were assessed using flow cytometric analysis on day 7 (*n* = 4) (O). Percentages of CD62L^+^CD44^+^ transferred CD8^+^ CTLs in the spleen were assessed on day 7 using flow cytometric analysis (*n* = 4) (P). Percentages of IFN‐γ^+^, TNF‐α^+^, IL‐2^+^, and Granzyme B^+^ (Gzm B) (Q); CD25^+^, CD27^+^, CD28^+^, CD44^+^, CD69^+^, and ICOS^+^ (R); PD‐1^+^, Tim‐3^+^, and CTLA‐4^+^ (S) transferred CD8^+^ CTL in the tumor were assessed on day 7 using flow cytometric analysis (*n* = 4). Data are presented as means ± SEM and were analyzed by two‐tailed unpaired Student's *t*‐test (N to S). All results are representative of at least three independent experiments. * *P* < 0.05, ** *P* < 0.01, *** *P* < 0.001, and **** *P* < 0.0001; ns, no statistically significant.

UA was initially identified as a microbial modulator that augments T cell activity in a concentration‐dependent manner using a Jurkat NFAT reporter cell line (Jurkat‐NFAT‐Luc) (Figure S1A,B, Supporting Information). Subsequent validation revealed that UA increased IL‐2 production at both mRNA and protein levels (Figure S1C, Supporting Information). Furthermore, UA dose‐dependently elevated IL‐2 production in primary CD8^+^ T cells from human peripheral blood mononuclear cells (PBMCs) and OT‐I TCR transgenic mice (**Figure** **1**A,B). Consistently, UA treatment enhanced TCR signaling in both human and mouse CD8^+^ T cells, as indicated by increased phosphorylation of PLCγ1, ZAP70, and LCK (Figure 1C; Figure S1D, Supporting Information). A cell viability assay demonstrated that a high dose of UA at 20 and 50 µm significantly decreased cell viability in antigen‐stimulated OT‐I CD8^+^ T cells (Figure 1D), suggesting that excessive UA might lead to overactivation‐induced cell death. Consequently, we selected a concentration of 10 µm to prevent cell death for subsequent studies. Specifically, compared with DMSO treatment, UA treatment increased the expression of IFN‐γ, TNF‐α, IL‐2, CD107a, and granzyme B in OT‐I CD8^+^ CTLs (Figure 1E,F; Figure S1E, Supporting Information) and enhanced the cytotoxic activity against antigen‐pulsed EL4 tumor cells in vitro (Figure 1G). Notably, adoptive transfer of UA‐treated OT‐Ι CD8^+^ CTLs significantly suppressed tumor growth in mice bearing subcutaneous EG7 tumors (ovalbumin‐expressing lymphoma EL4) (Figure 1H). Flow cytometric analysis showed that UA treatment significantly increased the accumulation of transferred CTLs in EG7 tumors (Figure 1I). Moreover, UA improved the antitumor activity of CTLs and increased their accumulation in blood and tumors in the lung metastasis model of B16‐MO5‐Fluc (luciferase‐expressing B16‐MO5) (Figure 1J–L). These findings suggest that UA has the potential to enhance adoptive transfer therapy.

Previous research has indicated that CD8^+^ T cells with central memory T (T_CM_) phenotype confer superior antitumor responses than effector memory T (T_EM_) cell subsets, primarily due to their enhanced replicative and persistence capacities.^[^ ^22^ ^]^ We observed that UA‐treated CTLs displayed a higher frequency of CD62L^+^CD44^+^ T_CM_ phenotype than DMSO‐treated CTLs (Figure S1F, Supporting Information). Co‐transfer experiments in naïve recipients revealed that UA‐treated CD8^+^ CTLs retained an elevated cell frequency after transfer (Figure S2A,B, Supporting Information), along with reduced apoptosis (Figure S2C, Supporting Information) and enhanced homeostatic proliferation in vivo (Figure S2D, Supporting Information). Similarly, in the subcutaneous B16‐MO5 tumor mouse model, UA treatment markedly increased the accumulation and persistence of transferred CTLs in the tumor on days 7 and 14 after cell transfer (Figure 1M,N; Figure S2E, Supporting Information). Consistently, UA treatment not only led to decreased apoptosis and increased proliferation of transferred CTLs in the tumor, spleen, and lymph node of recipient mice (Figure 1O; Figure S2F,G, Supporting Information) but also dramatically increased the proportion of CTLs with a CD62L^+^CD44^+^ T_CM_ phenotype (Figure 1P). The transferred CTLs pretreated with UA substantially enhanced the IFN‐γ, TNF‐α, IL‐2, and granzyme B‐producing capacity in the tumor, spleen, and lymph node (Figure 1Q; Figure S2H–J, Supporting Information). Furthermore, UA pretreatment increased the expression of activation markers (CD25, CD27, CD28, CD44, CD69, and ICOS) while reducing the expression of inhibitory receptors (PD‐1, Tim‐3, and CTLA4) on adoptively transferred CTLs in the tumor and spleen (Figure 1R,S; Figure S2K,L, Supporting Information), suggesting that UA treatment directly inhibits T cell exhaustion after adoptive transfer.

In summary, these results demonstrate that UA directly promotes the persistence and antitumor activity of CD8^+^ CTLs.

### 2.2 Urolithin A Regulates Mouse CD8^+^ CTL Function in an ERK Pathway‐Dependent Manner

**Figure 2:**
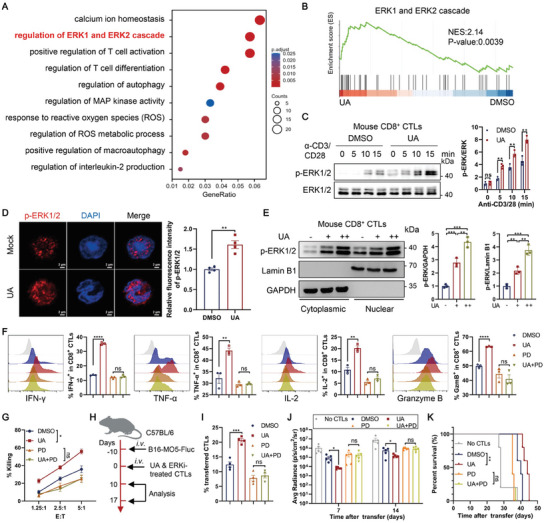
Urolithin A regulates mouse CD8^+^ CTL function through the ERK pathway. A,B) OT‐I CD8^+^ CTLs were treated with DMSO or 10 µm UA for 48 h and stimulated with anti‐CD3/28 (1 + 3 µg ml^−1^) antibodies for 6 hours, followed by RNA‐seq analysis. A) Scatter plot showing the Gene Ontology (GO) Biological Process enrichment results in the differentially expressed genes (DEGs) with RNA sequencing data of DMSO‐ and 10 µm UA‐treated CTLs. The dot size indicates the relative number of differentially expressed genes contained in the GO terms, and the shade of the dots indicates the adjusted *P* value (*P*adj) of the enrichment. RNA‐seq data are from one experiment with three technical replicates per sample. B) Gene set enrichment analysis (GSEA) plot of differentially expressed genes in ERK1 and ERK2 cascade pathway between DMSO‐ and UA‐treated CTLs. RNA‐seq data are from one experiment with three technical replicates per sample. C) OT‐I CD8^+^ CTLs were treated with DMSO and 10 µm UA for 48 h and stimulated with anti‐CD3/CD28 (1 + 3 µg ml^−1^) antibodies for indicated time points, followed by immunoblots analysis. Representative immunoblot image (left) and quantification (right, normalized to total ERK1/2) of p‐ERK1/2 in DMSO‐ and UA‐treated CTLs. Data are presented as means ± SEM (*n* = 3) and were analyzed by two‐tailed unpaired Student's *t*‐test. The immunoblots were performed three times independently. D) CD8^+^ CTLs were treated with DMSO or 10 µm UA for 48 h. The fluorescent images of CD8^+^ CTLs stained by p‐ERK1/2 (red) (left). White scale bar = 2 µm. Quantification of relative p‐ERK1/2 fluorescence intensity (right). Data are presented as means ± SEM (*n* = 3) and were analyzed by two‐tailed unpaired Student's *t*‐test. The experiments were performed three times independently. E) CD8^+^ CTLs were treated with DMSO or UA (+, 5 µm; ++, 10 µm) for 48 h and fractionated. Then, the cytoplasmic and nuclear protein fractions were blotted for p‐ERK1/2, GAPDH (cytoplasmic marker), or Lamin B1 (nuclear marker). Quantification of p‐ERK1/2 in the nucleus and cytoplasm (right). Data are presented as means ± SEM (*n* = 3) and were analyzed by two‐tailed unpaired Student's *t*‐test. The immunoblots were performed three times independently. F) OT‐I CD8^+^ T cells were treated with DMSO or UA (10 µm) in the presence or absence of PD0325901 (PD, 10 µm) for 48 h, followed by anti‐CD3/28 stimulation for 6 h. Expression of IFN‐γ, TNF‐α, IL‐2, and Granzyme B (Gzm B) in CD8^+^ T cells was assessed using flow cytometric analysis. Data are presented as means ± SEM (*n* = 3) and were analyzed by two‐tailed unpaired Student's *t*‐test. G) OT‐I CD8^+^ CTLs were treated with DMSO or UA (10 µm) in the presence or absence of PD0325901 (PD, 10 µm) for 48 h. Subsequently, cytotoxicity of the treated CD8^+^ CTLs against 10 nm OVA_257‐264_ peptide‐pulsed EL4 targets at indicated E:T ratios was determined in vitro. Data are presented as means ± SEM (*n* = 3) and were analyzed by two‐way ANOVA. H–K) OT‐I CTLs were treated with DMSO or UA (10 µm) in the presence or absence of PD0325901 (PD, 10 µm) for 48 h and transferred to B16‐MO5‐Fluc lung metastases‐bearing C57BL/6 mice on day 10 post‐inoculation. Schematic diagram of the adoptive transfer experiment (H). The percentage of transferred CTLs in the tumor was assessed using flow cytometric analysis (*n* = 4 mice per group) (I). Tumor growth is indicated by luciferase activity in the lung (*n* = 5 mice per group) (J). The survival curve was monitored (*n* = 5 mice per group) (K). Data are presented as means ± SEM and were analyzed by two‐tailed unpaired Student's *t*‐test (I, J) and Log‐rank test (K). All results are representative of at least three independent experiments. * *P* < 0.05, ** *P* < 0.01, and *** *P* < 0.001; ns, no statistically significant.

To elucidate how UA regulates CD8^+^ CTL function, we conducted RNA sequencing (RNA‐seq) on DMSO‐ and UA‐treated CD8^+^ CTLs. UA treatment resulted in differential expression of 945 transcripts, with 639 downregulated and 306 upregulated (1.5‐fold or more) (Figure S3A, Supporting Information). Gene ontology (GO) analysis of the RNA‐seq data revealed significant enrichment of biological processes associated with T cell activation and differentiation in UA‐treated CTLs (**Figure** **2**A), supporting UA's role as a T cell activator. Additionally, gene set enrichment analysis (GSEA) demonstrated the enrichment of signature genes related to T cell activation and the memory T cell pathway in UA‐treated CTLs (Figure S3B, Supporting Information).

Consistent with the GO analysis findings highlighting the regulation of the ERK1 and ERK1/2 cascade as a significantly enriched pathway (Figure 2A), GSEA confirmed the enrichment of the ERK1 and ERK2 cascade in UA‐treated CTLs (Figure 2B). Importantly, UA significantly enhanced ERK1/2 activation in OT‐I CTLs and primary human CD8^+^ T cells stimulated with anti‐CD3/CD28 antibodies, evidenced by increased phosphorylation of ERK1/2 at Tyr204/Thr202 (Figure 2C; Figure S3C, Supporting Information). Quantification using a commercial ELISA kit revealed a dose‐dependent increase in ERK1/2 phosphorylation in OT‐I CD8^+^ T cells treated with UA (Figure S3D, Supporting Information). Immunofluorescence staining further demonstrated an elevation of active ERK1/2 (pERK1/2), primarily localized in the nuclei, in UA‐treated CTLs (Figure 2D). Nucleocytoplasmic separation experiments confirmed that UA treatment increased p‐ERK in both the cytoplasm and the nucleus (Figure 2E), providing additional support for the stimulatory effect of UA on ERK1/2 activation.

To ascertain the involvement of the ERK pathway in UA‐mediated effects on CD8^+^ T cells, we treated CTLs with DMSO or UA in the presence of two ERK pathway inhibitors, PD0325901 (PD) and SCH772984 (SCH). Both ERK inhibitors nullified the UA‐enhanced cytokine‐producing capacity and cytotoxic activity against EL4 tumor in vitro (Figure 2F,G; Figure S3E,F, Supporting Information), indicating that UA regulates CD8^+^ CTL function through the ERK pathway. Consistently, in the presence of the ERK inhibitor PD0325901, UA failed to increase the frequency of transferred CTLs in the B16‐MO5 lung metastasis model (Figure 2H,I). Moreover, ERK inhibition eliminated the UA‐mediated enhancement of CTL adoptive transfer therapy in vivo, as demonstrated by bioluminescence imaging and survival curves (Figure 2J,K). In summary, both in vitro and in vivo studies suggest that UA treatment enhances the antitumor activities of mouse CD8^+^ CTLs through the ERK pathway.

### 2.3 Urolithin A Enhances the Antitumor Activities of Human CAR T Cells through the ERK Pathway

**Figure 3:**
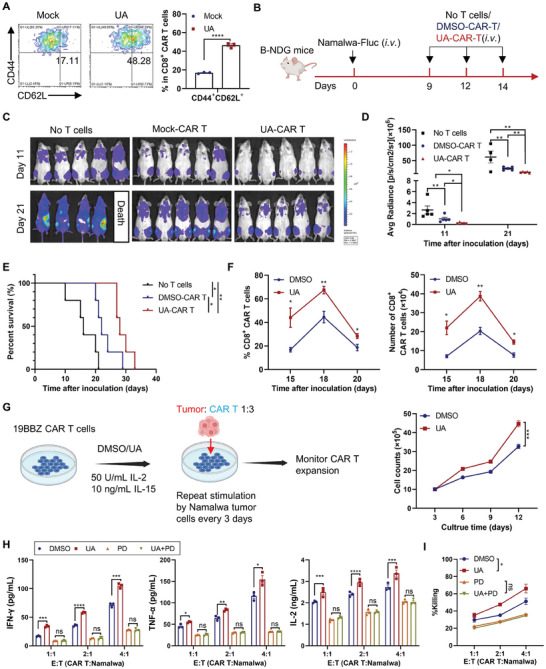
Urolithin A treatment enhances the antitumor activities of human CAR T cells through the ERK pathway. A) 19BBz CAR T cells were treated with DMSO or UA (10 µm) for 48 h. The percentage of CD62L^+^CD44^+^ CAR T cells was assessed using flow cytometric analysis. Data are presented as means ± SEM (*n* = 3) and were analyzed by two‐tailed unpaired Student's *t*‐test. B–F) Schematic diagram of adoptive transfer of DMSO or UA (10 µm)‐treated 19BBz CAR T cells into systemic Namalwa‐Fluc tumor‐bearing B‐NDG mice. Tumor growth was monitored using bioluminescence imaging. Representative images (C) and quantification (D) of bioluminescence and survival curves (E) were monitored (*n* = 5 mice per group). Percentage and number of transferred CD8^+^ CAR T cells (F) in peripheral blood from tumor‐bearing B‐NDG mice were evaluated using flow cytometry. Data are presented as means ± SEM and were analyzed by unpaired Student's *t*‐test (D, F) and Log‐rank test (E). G) Schematic diagram of induction of CAR T cell exhaustion (left). CAR T cells were treated with DMSO or UA (10 µm) for 48 h, followed by repeated stimulation with CD19‐expressing tumor cells (Namalwa) in vitro for 3 rounds. Cell counts of CAR T cells were determined by an automated cell counter after each stimulation. Data are presented as means ± SEM (*n* = 3) and were analyzed by two‐way ANOVA. H,I) 19BBz CAR T cells were treated with DMSO or UA (10 µm) in the presence or absence of ERK inhibitor PD0325901 (PD, 10 µm) for 48 h, followed by stimulated with Namalwa cells at E:T ratios of 1:1, 2:1 and 4:1 for 18 h. Cytokine production (IFN‐γ, TNF‐α, and IL‐2) of 19BBz CAR T cells in the supernatant was detected using ELISA (H). Cytotoxicity of 19BBz CAR T cells against Namalwa cells was determined in vitro using flow cytometry (I). Data are presented as means ± SEM (*n* = 3) and were analyzed by two‐tailed unpaired Student's *t*‐test (H) and two‐way ANOVA (I). All results are representative of at least three independent experiments. * *P* < 0.05, ** *P* < 0.01, *** *P* < 0.001 and ****P < 0.0001; ns, no statistically significant.

To explore whether UA exerts similar effects in human T cells, we generated 19BBz CAR T cells using human peripheral T cells^[^ ^23^ ^]^ and treated them with UA. UA did not affect CAR T cell viability at 10 µm (Figure S4A, Supporting Information) while significantly promoting the T_CM_ phenotype (CD44^+^CD62L^+^) (**Figure** **3**A). Notably, UA treatment markedly improved the in vivo antitumor activity of 19BBz CAR T cells in B‐NDG mice bearing Namalwa‐Fluc tumors, as evidenced by bioluminescence imaging and the survival curve (Figure 3B–E). UA pretreatment notably enhanced the persistence of transferred CAR T cells (Figure 3F), along with increased expression of genes such as *IL‐21*, *LEF1*, and *TCF7*, known to promote memory formation (Figure S4B, Supporting Information). In vitro repeated stimulation with CD19‐expressing tumor cells resulted in a more significant expansion of UA‐treated CAR T cells (Figure 3G).

In agreement with the enhanced in vivo antitumor activities, UA‐treated 19BBz CAR T cells exhibited increased production of IFN‐γ, TNF‐α, and IL‐2 at both protein and mRNA levels, along with enhanced cytotoxicity against CD19‐expressing human B lymphoblastoid cell lines Raji and Namalwa in vitro (Figure 3H,I; Figure S4C–E, Supporting Information). Importantly, ERK blockade with PD0325901 and SCH772984 inhibitors abolished UA‐enhanced cytokine production and cytotoxicity against Namalwa tumor cells (Figure 3H,I; Figure S4F,G, Supporting Information), confirming the universal role of the ERK pathway in UA‐mediated effects in both human and mouse T cells. Overall, our data suggest that UA treatment during the CAR T cell manufacturing process enhances CAR T cells' therapeutic potential and effector function through the ERK pathway.

### 2.4 Urolithin A Promotes MEK1/2‐Mediated ERK1/2 Activation by Directly Binding to ERK1/2

**Figure 4:**
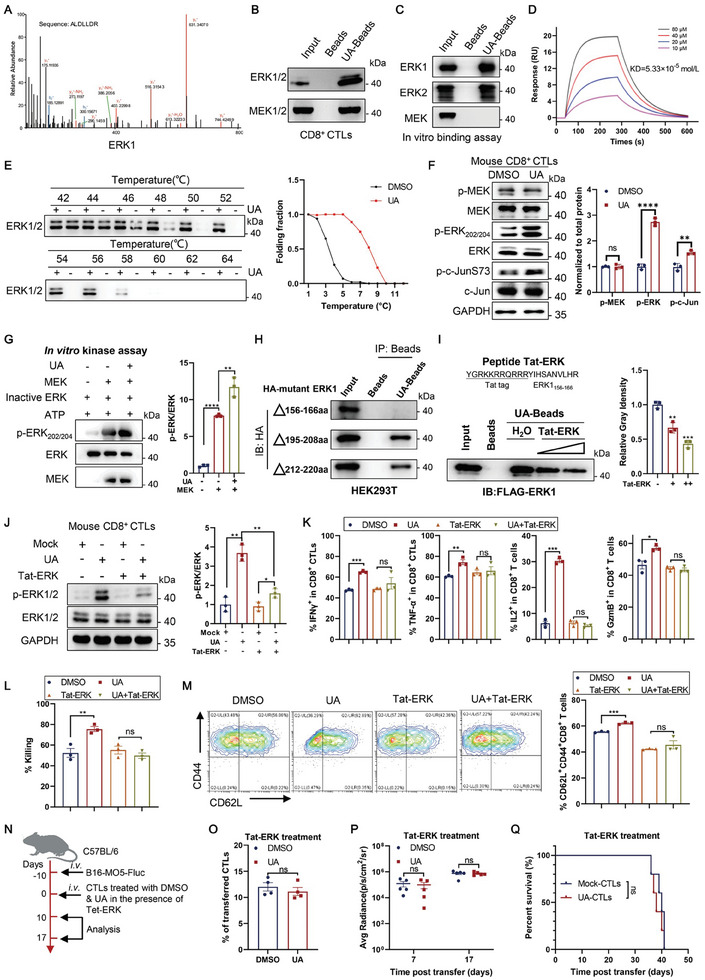
Urolithin A promotes ERK1/2 activation by directly binding to ERK1/2. A) UA‐conjugated Fe_3_O_4_ beads (UA‐Beads) were used to pull down target protein from Jurkat cells in vitro, followed by proteomic mass spectrometry analysis. Mass spectrometry results are from one experiment with three technical replicates per sample. B) Immunoblot analysis of ERK1/2 and MEK1 pulled down by UA‐beads from CD8^+^ CTL lysate. The pull‐down assay was repeated three times independently. C) In vitro binding assay of UA‐beads with ERK1, ERK2, and MEK1. Commercial purified Flag‐ERK1, Flag‐ERK2, and MEK1 proteins were incubated overnight with Fe3O4 control beads or UA‐beads at 4 °C. The proteins were then boiled with an SDS‐loading buffer and subjected to Western blot analysis. This experiment was repeated three times independently. D) SPR assay for the affinity between UA and purified his tagged ERK1 protein. SPR assay data are from one experiment with three technical replicates per sample. E) CTESA assay with UA‐treated CTLs. CD8^+^ CTLs were exposed to 10 µm UA (DMSO as control) for 24 h and heated at a specified temperature gradient (42° to 64 °C) for 3 min to denature the proteins, followed by Western blot analysis of ERK1/2. This experiment was repeated three times independently. F) CD8^+^ CTLs were treated with DMSO and UA (10 µm) for 48 h and stimulated with anti‐CD3/CD28 antibodies for 10 min, followed by immunoblot analysis of indicated proteins. Representative immunoblot image (left) and quantification of the indicated protein (right, normalized to total protein) are shown. Data are presented as means ± SEM (*n* = 3) and were analyzed by two‐tailed unpaired Student's *t*‐test. This experiment was repeated three times independently. G) Inactive ERK1/2 and activated MEK were incubated with UA (10 µm) and ATP for in vitro kinase assay. The reactions were subjected to immunoblot analysis with indicated antibodies. This experiment was repeated three times independently. Quantification analysis data are presented as means ± SEM (*n* = 3) and were analyzed by two‐tailed unpaired Student's *t*‐test. H) HEK293T cells were transfected with plasmids expressing the indicated HA‐tagged deletion mutant of ERK1 and then used to perform a pull‐down assay with control beads or UA‐beads. This experiment was repeated three times independently. I) Tat‐tagged ERK1_156‐166_ (Tat‐ERK) peptide (+, 5 µm; ++, 10 µm) was synthesized and used to compete for the pull‐down of commercial FLAG‐ERK1 with UA‐Beads. This experiment was repeated three times independently. Quantification analysis data are presented as means ± SEM (*n* = 3) and were analyzed by two‐tailed unpaired Student's *t*‐test. J) OT‐I CD8^+^ CTLs were treated with DMSO or UA (10 µm) in the presence or absence of Tat‐ERK (10 µm) for 48 h and stimulated with anti‐CD3/CD28 antibodies for 10 min, followed by immunoblot analysis of p‐ERK1/2 and ERK1/2. This experiment was repeated three times independently. Quantification of p‐ERK1(right, normalized to total ERK1/2) are presented as means ± SEM (*n* = 3) and were analyzed by two‐tailed unpaired Student's *t*‐test. K) OT‐I CTLs were treated with DMSO or UA (10 µm) in the presence or absence of Tat‐ERK (10 µm) for 48 h and then stimulated with anti‐CD3/CD28 antibodies for 6 h. Production of IFN‐γ, TNF‐α, IL‐2, and granzyme B in CD8^+^ CTLs was assessed using flow cytometric analysis. Data are presented as means ± SEM (*n* = 3) and were analyzed by two‐tailed unpaired Student's *t*‐test. This experiment was repeated three times independently. L) OT‐I CTLs were treated with DMSO or UA (10 µm) in the presence or absence of Tat‐ERK (10 µm) for 48 h. Cytotoxicity of the treated CTLs against 10 nm OVA_257‐264_ peptide‐pulsed EL4 targets was determined in vitro. Data are presented as means ± SEM (*n* = 3) and were analyzed by two‐tailed unpaired Student's *t*‐test. M) OT‐I CTLs were treated with DMSO or UA (10 µm) in the presence or absence of Tat‐ERK (10 µm) for 48 h. The percentage of CD62L^+^CD44^+^ CD8^+^ T cells was evaluated using flow cytometric analysis. Data are presented as means ± SEM (*n* = 3) and were analyzed by two‐tailed unpaired Student's *t*‐test. N–Q) OT‐I CTLs were treated with DMSO or UA (10 µm) in the presence of Tat‐ERK (10 µm) for 48 h and transferred into B16‐MO5‐Fluc lung metastases‐bearing C57BL/6 mice. Schematic diagram of the experiment (N). The percentage of transferred CTLs in the tumor was assessed using flow cytometric analysis (*n* = 4 mice per group) (O). Tumor growth is indicated by luciferase activity in the lung (*n* = 5 mice per group) (P). The survival curve was monitored (*n* = 5 mice per group) (Q). Data are presented as means ± SEM and were analyzed by two‐tailed unpaired Student's *t*‐test (O, P) and Log‐rank test (Q). All results are representative of at least three independent experiments. * *P* < 0.05, ** *P* < 0.01, and *** *P* < 0.001; ns, no statistically significant.

Given previous reports that UA acts as an Aryl hydrocarbon receptor (AhR) ligand,^[^ ^24^, ^25^, ^26^ ^]^ we initially investigated whether UA regulates CD8^+^ CTL function through AhR. Interestingly, the effects of UA on cytokine production in CD8^+^ CTLs were not reversed by the specific AhR inhibitor CH‐223191 (Figure S5A, Supporting Information),^[^ ^27^ ^]^ excluding AhR involvement in UA‐enhanced CD8^+^ T cell function. To identify UA's direct target protein in the ERK pathway that enhances T cell function, we conducted pull‐down assays using UA‐conjugated magnetic beads (UA‐beads) in Jurkat T cell lysates. Mass spectrometry analysis identified ERK1/2 and their upstream activator MEK1/2 in the ERK pathway as binding candidates of UA (**Figure** **4**A; DataS1, Supporting Information). The interaction of UA‐conjugated beads with ERK1/2 and MEK1/2 was confirmed by pull‐down experiments using UA‐beads in OT‐I CTL lysate (Figure 4B). Moreover, in vitro binding assays demonstrated that UA‐beads could directly bind to commercial purified ERK1 and ERK2 proteins but not MEK1 (Figure 4C), suggesting ERK1/2 as the direct binding target of UA. Consistently, competitive pull‐down assays with lysate from HEK293T cells overexpressing Flag‐ERK1 showed that unmodified free UA disrupted the pull‐down of Flag‐ERK1 with UA‐beads in a dose‐dependent manner (Figure S5B, Supporting Information). Surface plasmon resonance (SPR) assays with commercial ERK1 protein and UA showed that UA bound to ERK1 with a dissociation constant (*K*d) value of 53.3 µm (Figure 4D). Additionally, we confirmed the binding of UA to ERK1/2 in OT‐I CTLs with a cellular thermal shift assay (CETSA) (Figure 4E).

Since UA treatment did not affect the phosphorylation of ERK activator MEK1/2 but increased the phosphorylation of ERK substrate c‐Jun (Figure 4F), we hypothesized that UA might influence MEK1/2‐mediated ERK1/2 activation. In in vitro kinase assays with commercial ERK1 protein together with MEK1, UA markedly enhanced ERK1/2 activation (Figure 4G), demonstrating that UA enhances MEK‐mediated ERK1/2 activation. Our data collectively suggest that UA interacts with ERK1/2 and promotes their activation.

To understand the binding modes between UA and ERK1, we performed molecular docking studies using MDOCK. The results suggested that UA may bind to ERK1 in the region around amino acid residues 156–166 through hydrogen bond interactions, covering the conserved HRD component of the catalytic loop of the regulatory (R)‐spine (Figure S5C, Supporting Information).^[^ ^28^ ^]^ Consistently, the deletion of amino acid residues 156–166 (∆156‐166aa) from ERK1 completely disrupted the interaction between ERK1 and UA (Figure 4H). It has been reported that altered conformation of the R‐spine is vital in defining active and inactive states of ERK1/2.^[^ ^29^ ^]^ The molecular dynamic study indicates that UA binding to ERK1/2 (156‐166aa) might expose their activation sites (Figure S5D–F, Supporting Information). These data suggest that UA binding to ERK1 at the residue of R‐Spine might influence the interaction between MEK and ERK to activate ERK rather than directly activating MEK.

To further evaluate the functional role of UA binding to ERK1, we synthesized a fusion cell‐penetrating peptide containing the UA binding region (156‐166aa) of ERK with a trans‐activator of transcription (Tat) tag positioned at its N‐terminal region (Tat‐ERK_156‐166_, YQRKKRRQRRRYIHSANVLHR), which was proved to inhibit the interaction of UA with ERK1 (Figure 4I). As expected, Tat‐ERK_156‐166_ efficiently blocked the UA‐enhanced ERK1/2 activation and showed no apparent cytotoxicity to CD8^+^ CTLs at 1 to 50 µm (Figure 4J; Figure S5G, Supporting Information). We also observed that Tat‐ERK_156‐166_ inhibited the cytokine production and cytotoxicity of UA‐treated OT‐I CTLs in a dose‐dependent manner (Figure S5H,I, Supporting Information). Interestingly, Tat‐ERK_156‐166_ at 10 µm abrogated the enhanced cytokine production and cytotoxic capacity induced by UA (Figure 4K,L). Moreover, in the presence of Tat‐ERK_156‐166_, UA failed to promote memory formation, as revealed by the loss of CD62L induction (Figure 4M). When we transferred OT‐Ι CD8^+^ CTLs, which were pretreated with UA or DMSO in combination with Tat‐ERK_156‐166_, into B16MO5‐Fluc lung metastases 10 days after tumor inoculation (Figure 4N), there were no significant differences in the accumulation of transferred CTLs, tumor progression, and survival time between the two groups (Figure 4O–Q). Thus, our results demonstrate that UA activates ERK1/2 by directly binding to aa156‐166 on ERK1, thereby enhancing the antitumor activity and persistence of CD8^+^ CTLs.

### 2.5 UA‐Primed ERK1/2 Triggers ULK1 Activation and Downstream Autophagy Flux to Promote CD8^+^ T Cell Function

**Figure 5:**
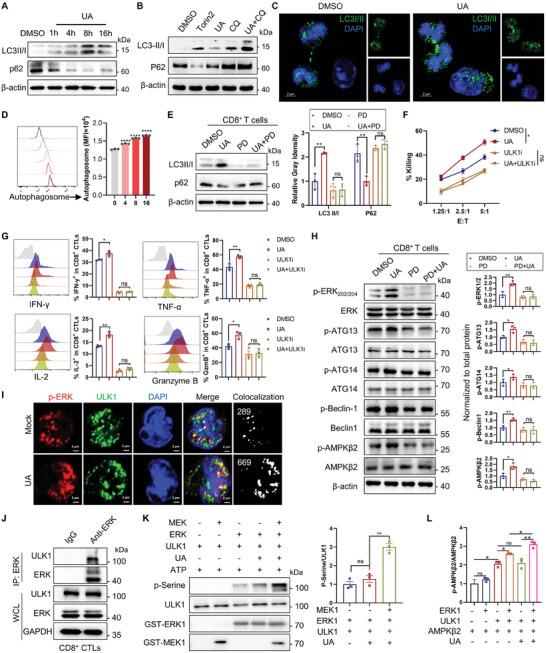
Urolithin A‐primed ERK1/2 triggers ULK1 activation and downstream autophagy flux to promote CD8^+^ T cell function. A) OT‐I CD8^+^ CTLs were treated with 10 µm UA or DMSO for 1, 4, 8, or 16 h, followed by immunoblot analysis. A representative image from three independent experiments is shown. B) OT‐I CD8^+^ CTLs were treated with 10 µm UA or DMSO for 48 h in the presence or absence of 10 µm chloroquine (CQ) for the last 4 h, followed by Western blot analysis. Torin2 (10 µm), an autophagy inducer, was used as a positive control. This experiment was repeated three times independently. C) Representative images of LC3I/II staining in OT‐I CD8^+^ T cells treated with 10 µm UA or DMSO for 48 h. The immunofluorescence images show LC3I/II (green) and DAPI (blue) in CTLs. White scale bars, 2 µm. This experiment was repeated three times independently. D) OT‐I CD8^+^ CTLs were treated with 10 µm UA or DMSO for 4, 8, and 16 h, followed by flow cytometric analysis of autophagosome. Representative flow cytometric histograms are shown (left). The mean fluorescence intensity (MFI) of autophagosome (right) are presented as means ± SEM (*n* = 3) and were analyzed by two‐tailed unpaired Student's *t*‐test. This experiment was repeated three times independently. E) OT‐I CD8^+^ CTLs cells were treated with 10 µm UA or DMSO in the presence or absence of ERK inhibitor PD0325901 (PD, 10 µm) for 48 h, followed by Western blot analysis. Representative immunoblot image (left) and quantification of the indicated protein (right, normalized to β‐actin) are shown. Data are presented as means ± SEM (*n* = 3) and were analyzed by two‐tailed unpaired Student's *t*‐test. This experiment was repeated three times independently. F) OT‐I CD8^+^ CTLs were treated with 10 µm UA or DMSO for 48 h in the presence or absence of ULK1 inhibitor MRT68921 (5 µm) for the last 4 h. Subsequently, cytotoxicity of the treated CD8^+^ CTLs against 10 nm OVA_257‐264_ peptide‐pulsed EL4 targets was determined in vitro at indicated E:T ratios. Data are presented as means ± SEM (*n* = 3) and were analyzed by two‐way ANOVA. G) OT‐I CD8^+^ CTLs were treated with 10 µm UA or DMSO for 48 h in the presence or absence of ULK1 inhibitor MRT68921 (5 µm) for the last 4 h and then stimulated with anti‐CD3/28 for 6 h. Production of IFN‐γ, TNF‐α, IL‐2, and granzyme B (Gzm B) in CD8^+^ CTLs was assessed using flow cytometric analysis. Data are presented as means ± SEM (*n* = 3) and were analyzed by two‐tailed unpaired Student's *t*‐test. This experiment was repeated three times independently. H) OT‐I CD8^+^ CTLs cells were treated with 10 µm UA or DMSO in the presence or absence of ERK inhibitor PD0325901 (PD, 10 µm) for 48 h, followed by Western blot analysis. Representative immunoblot image (left) and quantification of the indicated protein (right, normalized to total protein) are shown. Data are presented as means ± SEM (*n* = 3) and were analyzed by two‐tailed unpaired Student's *t*‐test. This experiment was repeated three times independently. I) OT‐I CTLs were treated with 10 µm UA or DMSO for 48 h, followed by anti‐CD3/28 stimulation for 10 min. The co‐localization of phosphorylated ERK1/2 (p‐ERK1/2) and ULK1 was assessed by immunofluorescence staining analysis. Immunofluorescence images show p‐ERK (red), ULK1 (green), and DAPI (blue) in CTLs. White scale bars, 2 µm. This experiment was repeated three times independently. J) Co‐immunoprecipitation analysis of endogenous ERK1/2 and ULK1 using ERK1/2 antibody in OT‐I CTLs. This experiment was repeated three times independently. K) Purified ULK1 and ERK1 were incubated with MEK and ATP in the presence or absence of UA (10 µm) for in vitro kinase assay. The reactions were subjected to immunoblot analysis with indicated antibodies. Representative immunoblot image (left) and quantification of the serine phosphorylation of ULK1 (right, normalized to total ULK1) are shown. Quantification data from three independent experiments are presented as means ± SEM (*n* = 3) and were analyzed by two‐tailed unpaired Student's *t*‐test. L) Purified ULK1 and AMPKβ2 were incubated with ERK1 and ATP in the presence or absence of UA (10 µm) for in vitro kinase assay. The reactions were subjected to immunoblot analysis with indicated antibodies. Quantification of p‐AMPKβ2 (normalized to total AMPKβ2) from three independent experiments are presented as means ± SEM (*n* = 3) and were analyzed by two‐tailed unpaired Student's *t*‐test. All results are representative of at least three independent experiments. * *P* < 0.05, ** *P* < 0.01, and *** *P* < 0.001; ns, no statistically significant.

Although UA has been reported to regulate mitophagy and autophagy,^[^ ^15^, ^16^, ^19^ ^]^ whether the ERK pathway is involved in this process is unknown. In agreement with GO analysis of RNA‐seq data showing the enrichment of pathways related to the regulation of autophagy and positive regulation of macroautophagy in UA‐treated CTLs (Figure 2A), Western blot assay showed a significant increase of LC3‐II, a biomarker of autophagy, accompanied by a reduction of autophagy receptor p62 in CD8^+^ CTLs upon UA treatment (**Figure** **5**A; Figure S6A, Supporting Information). However, this phenomenon was reversed in the presence of chloroquine (CQ), an autophagic inhibitor that impairs autophagosome‐lysosome fusion (Figure 5B; Figure S6B, Supporting Information). Furthermore, confocal microscopy imaging showed that UA treatment dramatically increased LC3 and ATG16‐positive puncta in CD8^+^ CTLs (Figure 5C; Figure S6C, Supporting Information). Flow cytometry with the CytoID dye staining consistently displayed the increased formation of endogenous autophagosomes in UA‐treated CTLs (Figure 5D). In agreement with previous studies reporting the role of UA in mitophagy,^[^ ^15^ ^]^ UA treatment reduced Tom20 expression in CTLs (Figure S6D, Supporting Information). Notably, UA failed to induce autophagy in the presence of ERK inhibitor PD0325901 (Figure 5E). In the CTL adoptive transfer therapy model, ERK blockade with both ERK inhibitor and Tat‐ERK_156‐166_ eliminated the UA‐enhanced autophagosome in CTLs (Figure S6E, Supporting Information), implying that UA regulates autophagy through the ERK signals. Furthermore, pretreatment with several autophagy inhibitors, including MRT68921 (inhibitor of ULK1, ULKi), CQ, and 3‐Methyladenine (3‐MA), completely abolished the augmented killing ability and cytokine production elicited by UA in CD8^+^ CTLs (Figure 5F,G; Figure S6F,G, Supporting Information). The above data demonstrate that UA‐activated ERK1/2 triggers autophagy flux to enhance CD8^+^ CTL function.

ULK1 complex is located upstream of the autophagy pathway, and its kinase activity is essential for autophagy initiation.^[^ ^30^ ^]^ Building on the observation that the ULK1 inhibitor disrupted UA‐enhanced cytokine production, we hypothesized that UA‐activated ERK1/2 might regulate autophagy by activating ULK1. To validate this hypothesis, CD8^+^ T cells were treated with UA or DMSO in the presence or absence of the ERK inhibitor PD0325901. UA treatment enhanced ULK1 activity, as evidenced by the increased phosphorylation of its substrates, including Beclin1, ATG13, ATG14, and AMPKβ2 (Figure 5H).^[^ ^31^ ^]^ In contrast, the ERK inhibitor abrogated UA‐induced ULK1 activation, suggesting that UA enhanced ULK1 activation via ERK1/2.

Since ERK1/2 are serine/threonine kinases and regulate ULK1 activation, we further investigated whether ULK1 is the direct substrate of ERK1/2. Immunofluorescence staining demonstrated the colocalization of ULK1 with both ERK and p‐ERK in primary CD8^+^ T cells, and this colocalization was enhanced by UA treatment (Figure 5I; Figure S6H, Supporting Information). Additionally, UA significantly increased ULK1 puncta formation in CD8^+^ T cells (Figure 5I; Figure S6H, Supporting Information). Consistently, the immunoprecipitation assay confirmed the interaction between ERK1/2 and FLAG‐ULK1 in HEK293T cells and endogenous ULK1 in CD8^+^ CTLs (Figure 5J; Figure S6I, Supporting Information). Furthermore, the in vitro kinase assay showed that UA, in conjunction with MEK1 and ERK1, facilitated the serine phosphorylation of commercial Flag‐ULK1 (Figure 5K). Additionally, the co‐presence of UA and purified active ERK1, but not UA alone, augmented the ULK1‐mediated phosphorylation of purified AMPKβ2 (Figure 5L; Figure S6J, Supporting Information). Collectively, our findings indicate that UA‐mediated ERK1/2 activation promotes ULK1 phosphorylation and kinase activity, initiating autophagy in CD8^+^ CTLs.

### 2.6 The UA‐ERK1/2‐ULK1 Cascade‐Mediated Autophagy Promotes Cellular Metabolic Adaption and Restricts ROS to Maintain T Cell Function

Autophagy has been reported to drive T cells' metabolic reprogramming, promoting persistence potential.^[^ ^32^, ^33^, ^34^, ^35^ ^]^ Correspondingly, GSVA and GO analysis of RNA‐seq data displayed the enrichment of metabolic‐related pathways in UA‐treated T cells, including oxidative phosphorylation, glycolysis/gluconeogenesis, and the pyruvate metabolism pathway (Figure S7A,B, Supporting Information). Heatmap analysis revealed that genes related to OXPHOS, glycolysis, and fatty acid synthesis, including *Atp5a1*, *Cox5a*, *Mtcon2, Ndufa12, Slc2a2*, and *Pgam1*, were up‐regulated in UA‐treated CTLs (Figure S7C, Supporting Information), indicating an increased metabolic activity in these cells.

**Figure 6:**
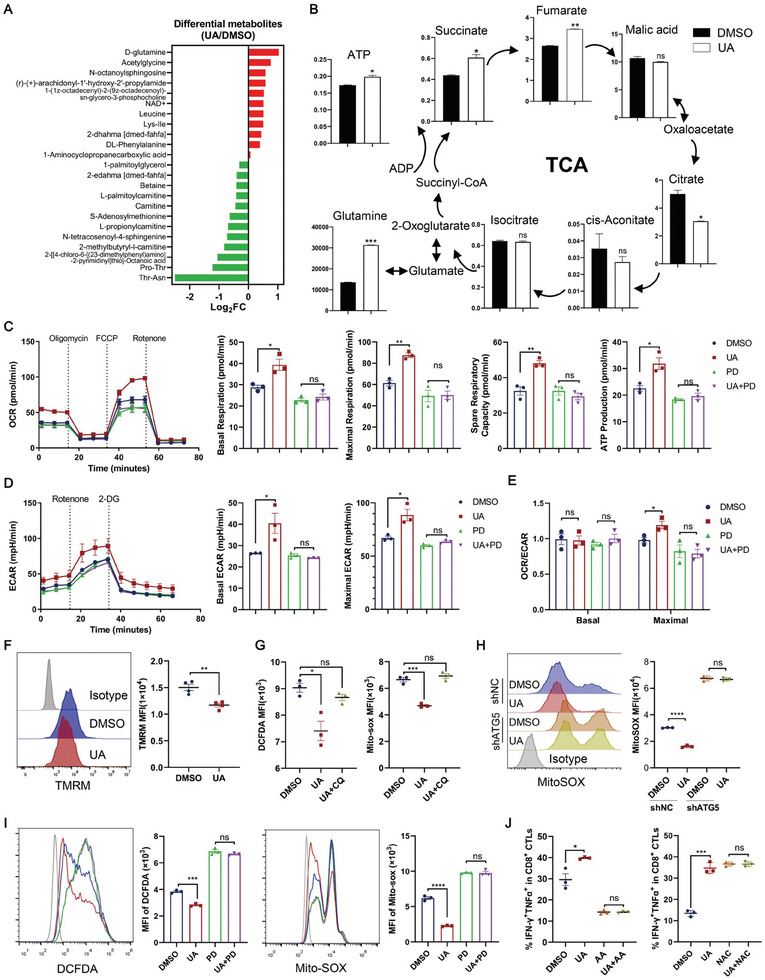
UA‐ERK1/2‐ULK1 cascade‐mediated autophagy regulates cellular metabolism and ROS levels to maintain T cell function. A,B) OT‐I CD8^+^ CTLs cells were treated with 10 µm UA or DMSO for 48 h. Metabolic intermediates were analyzed by mass spectrometry. Differential metabolites (A) and metabolic intermediates in the tricarboxylic acid (TCA) cycle (B) between DMSO‐ and UA‐treated CTLs are shown. Data are presented as means ± SEM (*n* = 3) and were analyzed by two‐tailed unpaired Student's *t*‐test (B). The data are from one experiment with three technical replicates per sample. C) OT‐I CD8^+^ CTLs cells were treated with 10 µm UA or DMSO in the presence or absence of ERK inhibitor PD0325901 (PD, 10 µm) for 48 h. Oxygen consumption rate (OCR) was measured in treated CD8^+^ CTLs under basal and stimulated conditions with oligomycin, FCCP, and rotenone. Basal respiration, maximal respiration, spare respiratory capacity statistics, and ATP production were shown. Data are presented as means ± SEM (*n* = 3) and were analyzed by two‐tailed unpaired Student's *t*‐test. D) OT‐I CD8^+^ CTLs cells were treated with 10 µm UA or DMSO in the presence or absence of ERK inhibitor PD0325901 (PD, 10 µm) for 48 h. Extracellular acidification rate (ECAR) of the treated OT‐I CTLs was measured under basal and stimulated conditions with glucose, rotenone, and 2‐deoxy‐glucose (2‐DG). Basal and maximal ECAR were shown. Data are presented as means ± SEM (*n* = 3) and were analyzed by two‐tailed unpaired Student's *t*‐test. E) Ratios of OCR to ECAR in DMSO‐ and UA‐treated CD8^+^ CTLs were shown. Data are presented as means ± SEM (*n* = 3) and were analyzed by two‐tailed unpaired Student's *t*‐test. F) OT‐I CD8^+^ CTLs were treated with 10 µm UA or DMSO for 48 h, followed by determination of TMRM staining using flow cytometric analysis. Data are presented as means ± SEM (*n* = 3) and were analyzed by two‐tailed unpaired Student's *t*‐test. G) OT‐I CD8^+^ CTLs were treated with 10 µm UA or DMSO for 48 h in the presence or absence of 10 µm chloroquine (CQ) for the last 4 hours, followed by flow cytometric analysis of ROS (DCFDA) (left) and mitochondrial superoxide (Mito‐sox) (right). Data are presented as means ± SEM (*n* = 3) and were analyzed by two‐tailed unpaired Student's *t*‐test. H) OT‐I CD8^+^ CTLs transduced with control, or ATG5 shRNA, were treated with 10 µm UA or DMSO for 48 h, followed by flow cytometric analysis of mitochondrial superoxide (Mito‐sox). Data are presented as means ± SEM (*n* = 3) and were analyzed by two‐tailed unpaired Student's *t*‐test. I) OT‐I CD8^+^ CTLs were treated with 10 µm UA or DMSO in the presence or absence of ERK inhibitor PD0325901 (PD, 10 µm) for 48 h, followed by flow cytometric analysis of ROS (DCFDA) and mitochondrial superoxide (Mito‐sox). Data are presented as means ± SEM (*n* = 3) and were analyzed by two‐tailed unpaired Student's *t*‐test. J) OT‐I CD8^+^ CTLs cells were treated with 10 µm UA or DMSO in the presence or absence of antimycin A (AA, 0.04 µm) or NAC (2.5 mm) for 48 h, followed by flow cytometric analysis of IFN‐γ and TNF‐α. Data are presented as means ± SEM (*n* = 3) and were analyzed by two‐tailed unpaired Student's *t*‐test. All results are representative of at least three independent experiments. * *P* < 0.05, ** *P* < 0.01, *** *P* < 0.001, and **** *P* < 0.0001; ns, no statistically significant.

Metabolomics analysis was performed on UA‐treated CD8^+^ CTLs and control cells to assess metabolic changes. A total of 769 metabolites were detected, with 309 up‐regulated and 172 down‐regulated (1.5‐fold or more) (Figure S7D, Supporting Information). In line with a previous study^[^ ^36^ ^]^ demonstrating that ERK/MAPK regulates glutamine metabolism during activation and our data mentioned above indicating increased ERK activation in UA‐treated CD8^+^ CTLs, D‐glutamine was the top 1 increased metabolites in UA‐treated CTLs (**Figure** **6**A). KEGG analysis of differentiated metabolites highlighted central carbon metabolism in cancer as one of the top enriched altered metabolite pathways in UA‐treated CD8^+^ CTLs compared with DMSO‐treated CTLs (Figure S7E, Supporting Information). Subsequent energy metabolomics revealed significant enrichment of succinate and fumarate, metabolites involved in the tricarboxylic acid (TCA) cycle, in UA‐treated CD8^+^ CTLs (Figure 6B). However, UA treatment significantly reduced citrate, associated with *de novo* fatty acid synthesis (Figure 6B), aligning with RNA‐seq data showing increased expression of genes related to fatty acid synthesis (Figure S7C, Supporting Information). Targeted energy metabolomics also indicated a significant increase in glutamine levels in UA‐treated CD8^+^ CTLs (Figure 6B), supporting the idea that glutamine contributes to fatty acid synthesis and replenishes TCA intermediates that promote oxidative phosphorylation.

Notably, Seahorse analysis revealed that UA treatment increased cellular metabolism in CD8^+^ T cells, enhancing their survival and function. UA‐treated CTLs exhibited increased oxygen consumption rate (OCR) (Figure 6C) and glycolysis, measured by extracellular acidification rate (ECAR) (Figure 6D). The ratios of OCR to ECAR increased under stress (Figure 6E), indicating UA actively reprograms T cell metabolism toward higher oxidative phosphorylation (OXPHOS), providing energy for T cell proliferation and activation. Additionally, ATP production in UA‐treated CTLs was significantly higher than in control DMSO‐treated cells (Figure 6C, right panel). Importantly, both ERK and ULK1 inhibitors abolished the aforementioned UA‐induced metabolic changes (Figure 6C–E; Figure S8A–C, Supporting Information), suggesting that UA regulates the metabolic adaption of CD8^+^ CTLs through the ERK‐ULK1 axis. Furthermore, UA‐treated CD8^+^ CTLs exhibited a metabolic feature that enhanced T cell stemness for cancer therapy,^[^ ^37^ ^]^ displaying an enhanced spare respiratory capacity (Figure 6C) and reduced mitochondrial membrane potential (Figure 6F).

We further assessed global differences at the mRNA level to gain further insights into how UA regulates cellular metabolism to enhance functions in CTLs. Notably, GSEA identified significant enrichment of reactive oxygen species pathways in DMSO‐treated CTLs compared to UA‐treated CTLs (Figure S8D, Supporting Information), indicating a UA‐mediated increased capacity for reducing oxidative stress, a major function of autophagy.^[^ ^38^ ^]^ Flow cytometric analysis revealed that UA treatment decreased cellular and mitochondrial ROS in CD8^+^ CTLs in an ERK‐ULK1‐autophagy axis‐dependent manner, as evidenced by the results using the autophagy inhibitor CQ and ATG5 silencing, as well as ERK and ULK1 inhibitors (Figure 6G–I; Figure S8E, Supporting Information). Furthermore, the cytokine production in CD8+ CTLs was suppressed by the ROS inducer arachidonic acid (AA), whereas it was augmented by the ROS scavenger N‐acetylcysteine (NAC) (Figure 6J). It is noteworthy that UA failed to enhance cytokine production of CD8+ CTLs when either AA or NAC was present (Figure 6J), suggesting that UA promoted cytokine‐producing ability through modulating ROS.

Taken together, these data confirm that UA enhances cellular metabolic fitness and maintains ROS homeostasis through autophagy, thereby modulating the effector function of CD8^+^ CTLs.

### 2.7 Oral Urolithin A at Low Dose Promotes Antitumor Immune Responses

**Figure 7:**
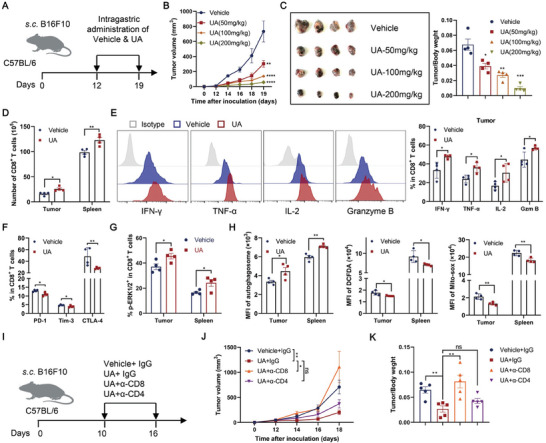
Oral administration of Urolithin A promotes antitumor immune responses. A–C) Schematic diagram of subcutaneous B16F10 tumor models treated with daily intragastric administration of UA at different doses (50, 100, and 200 mg kg^−1^, sunflower oil as vehicle control) from day 12 to 19 (A). Tumor growth curves (B) and tumor‐to‐body weight ratios on day 20 (C) were monitored (*n* = 4 mice per group). Data are presented as means ± SEM and were analyzed by two‐way ANOVA (B) and two‐tailed unpaired Student's *t*‐test (C). D–H) Subcutaneous B16F10 tumor models were treated with daily intragastric administration of UA at 50 mg kg^−1^ (sunflower oil as vehicle control) from day 12 to 19. Number of CD8^+^ T cells in the tumor and spleen were assessed using flow cytometric analysis (D, *n* = 4 mice per group). Percentages of IFN‐γ^+^, TNF‐α^+^, IL‐2^+^, and Granzyme B^+^ tumor‐infiltrating CD8^+^ T cells were estimated by flow cytometric intracellular staining (E, *n* = 4 mice per group). PD‐1, Tim‐3, and CTLA‐4 expression on CD8^+^ T cells were assessed using flow cytometry (F, *n* = 4 mice per group). Flow cytometric analysis of p‐ERK1/2 in tumor‐infiltrating and splenic CD8^+^ T cells was shown (G, *n* = 4 mice per group). Autophagosome, DCFDA, and Mito‐sox staining in CD8^+^ T cells from the tumor and spleen were assessed using flow cytometric analysis (H, *n* = 4 mice per group). All data are presented as means ± SEM and were analyzed by two‐tailed unpaired Student's *t*‐test. I–K) Schematic diagram of subcutaneous B16F10 tumor models treated with daily intragastric administration of UA at 50 mg kg^−1^ in the presence of anti‐CD4 or CD8 antibodies from day 10 to 16 (I). Tumor volumes (J) and tumor‐to‐body weight ratios on day 17 (K) were monitored (*n* = 5 mice per group). Data are presented as means ± SEM and were analyzed by two‐way ANOVA (J) and two‐tailed unpaired Student's *t*‐test (K). All results are representative of at least three independent experiments. * *P* < 0.05, ** *P* < 0.01, *** *P* < 0.001, and **** *P* < 0.0001; ns, no statistically significant.

In a recent clinical trial demonstrating the safety and bioavailability of UA, a low dose of 250 mg was administered,^[^ ^19^ ^]^ equivalent to 50 mg kg^−1^ in mice. We conducted oral administration of UA at doses of 50, 100, and 200 mg kg^−1^ in subcutaneous B16F10 tumor‐bearing mice starting from day 12 post‐tumor inoculation (**Figure** **7**A). Importantly, none of these UA doses induced any abnormal changes in body weight or the weight of various organs, including the heart, liver, spleen, lung, and kidney (Figure S9A,B; Supporting Information). Histological examination through H&E staining revealed no apparent abnormalities (Figure S9C; Supporting Information). Notably, oral UA administration dose‐dependently inhibited tumor progression and reduced tumor burden (Figure 7B,C). Subsequent analysis of the impact of oral UA dosing on immune cells in the subcutaneous B16F10 tumor model demonstrated an increase in the number of CD8^+^ T cells in both the tumor and spleen Figure 7D; Figure S11A, Supporting Information), substantiating that UA promotes CD8^+^ T cell persistence. CD8^+^ T cells from UA‐treated mice exhibited enhanced production of IFN‐γ, TNF‐α, IL‐2, and granzyme B (Figure 7E; Figure S11B, Supporting Information) and downregulated expression of PD‐1, Tim‐3, and CTLA‐4 (Figure 7F). Moreover, consistent with our in vitro findings, both tumor‐infiltrating and splenic CD8^+^ T cells from UA‐treated tumor‐bearing mice displayed increased phosphorylation of ERK1/2 and autophagy, along with lower ROS levels (Figure 7G,H), indicating that oral UA administration continued to enhance ERK1/2 activation and autophagy, thereby reducing ROS within CD8^+^ T cells in vivo. UA treatment in vivo did not affect the abundances of CD4^+^ T cells, regulatory T cells (Tregs), and NK cells while decreasing the infiltration of myeloid‐derived suppressor cells (MDSCs) in the tumor and spleen (Figure S11C–E, Supporting Information). UA did not impact the cytokine‐producing ability in NK cells from UA‐treated mice (Figure S11F,G, Supporting Information). Depletion of CD8^+^ T cells, but not CD4^+^ T cells, with antibodies abrogated the antitumor effect of UA (Figure 7I–K), suggesting that the UA‐enhanced anti‐tumor response is CD8^+^ T cell‐dependent. Additionally, we confirmed the therapeutic and immunoregulatory role of UA in mice bearing B16F10 lung metastases and subcutaneous MC38 tumors, which were treated with UA for 7 days (Figure S12, Supporting Information). Recent studies have reported that UA administration at a higher dose of 2.28 g kg^−1^ demonstrated antitumor effects in murine colorectal cancer.^[^ ^21^ ^]^ Our current findings further substantiate the therapeutic potential of UA, as even at lower doses, direct administration of UA proves to be beneficial for antitumor immunity.

## 3 Discussion

Recent studies have highlighted the pivotal role of the microbial metabolite UA in immune system regulation.^[^ ^39^ ^]^ Notably, UA has been identified as a promoter of CD8^+^ T memory stem cell generation, enhancing the antitumor activity.^[^ ^21^ ^]^ To comprehensively understand UA‐mediated effects on T cell effector function, we investigated its impact and identified ERK1/2 as a direct UA target protein. Our findings demonstrated that UA, through binding and activating ERK1/2, could enhance both the persistence and effector function of primary CD8^+^ CTLs and human CAR T cells, even at low doses. This underscores the promising potential of UA in T cell‐based tumor immunotherapy.

Our study investigated the mechanisms through which UA augments persistence and antitumor activity in CD8^+^ CTLs. Intriguingly, UA enhances TCR signaling and activates NFAT, leading to increased production of autocrine IL‐2 and effector molecules. Simultaneously, UA directly binds to ERK1/2 kinases, enhancing their activation and modulating LCK to boost TCR signaling.^[^ ^40^ ^]^ Both NFAT and ERK1/2 kinases play pivotal roles in T cell activation and survival. It has been established that the formation of activating NFAT‐AP‐1 complexes is essential for preventing T cell exhaustion, enhancing long‐term proliferative capacity, and promoting proper antitumor activity.^[^ ^41^ ^]^ However, if AP‐1, the downstream transcription factor of ERK,^[^ ^42^ ^]^ is absent, NFAT alone induces T cell anergy and exhaustion.^[^ ^43^ ^]^ Therefore, UA‐induced ERK1/2 activation may facilitate the formation of the NFAT‐AP1 complex, sustaining proper persistence and preventing T‐cell exhaustion.

While UA is a well‐known small molecule regulating autophagy, mitophagy and mitochondrial function,^[^ ^44^ ^]^ its direct target remains unknown. Although UA has been reported to be an Aryl hydrocarbon receptor (AhR) ligand,^[^ ^24^, ^25^, ^26^ ^]^ we showed that UA promotes CTLs antitumor functions in an AhR‐independent manner. Our study provides hitherto undocumented evidence that UA directly binds to ERK1/2, and the interaction site on ERK1 was identified. A small peptide, Tat‐ERK_156‐166_, designed to target this site, significantly blocked the specific binding. This peptide disrupted the UA‐enhanced memory potential and effector function, confirming the crucial role of the direct interaction between UA and ERK1/2 in CTL antitumor immunity. We also discovered that UA‐induced activation of ERK1/2 leads to the activation of ULK1, initiating autophagy, including mitophagy,^[^ ^30^, ^45^, ^46^, ^47^ ^]^ consistent with previous reports showing that activated ERK1/2 potentiates the catalytic activity of cytoplasmic proteins.^[^ ^48^ ^]^ Our findings indicate that UA‐activated ERK1/2, localized in both the nuclei and cytoplasm, is essential for interaction with ULK1, proposing a novel ERK1/2‐ULK1 cascade in UA‐induced autophagy in CD8^+^ T cells.

The UA‐ERK1/2‐ULK1 cascade plays a crucial role in promoting the autophagic flux in CD8^+^ CTLs, leading to the reduction of oxidative stress and facilitating metabolic adaptation for the maintenance of T cell fitness. ERK1/2 and ULK1, being widely expressed, hold critical functions in various cell types, including T and tumor cells. The activation of ERK1/2 in tumor cells can either stimulate tumor growth or induce apoptosis, depending on its subcellular localization.^[^ ^48^ ^]^ In contrast, UA has been reported to directly inhibit tumor growth in vitro,^[^ ^49^ ^]^ implying that UA‐induced ERK1/2 activation might be cell‐type dependent. A recent paper demonstrated that ERK1/2 phosphorylation in glioblastoma predicts the survival of patients following anti‐PD‐1 immunotherapy, highlighting ERKs as effective immunotherapy targets.^[^ ^50^ ^]^ Investigating the role of the UA‐ERK1/2‐ULK1 axis and the usage of the small peptide Tat‐ERK_156‐166_ in different cell types would be intriguing.

In the previously published paper on UA's effects on CD8^+^ T cells, it was suggested that UA did not influence T_CM_ differentiation.^[^ ^21^ ^]^ However, our study reveals that UA not only promotes T_CM_ differentiation but also enhances T cell activation and effector function in both murine CD8^+^ CTLs and human CAR T cells. The discrepancy between our results and the previous study may be attributed to differences in UA doses and experimental settings. The prior studies used UA at 25 and 50 µm for in vitro treatment, leading to the inhibition of antigen‐induced expansion of primary OT‐I CD8^+^ T cells. Excessive UA might cause elevated cell death in primary CD8^+^ T cells, resulting in effector T cell death and T_SCM_ accumulation. In our study, a lower UA dose (10 µm) was employed, preserving cell viability while enhancing the superior antitumor activity of CTLs and CAR T cells. Moreover, the UA dose in our in vivo study was lower than in other studies, suggesting that low‐dose UA is advantageous for antitumor immunity, positioning UA as a convenient and cost‐effective agent for tumor therapy. Further investigation is needed to determine the antitumor activity of UA at different doses in various preclinical models.

Notably, only ≈40% of elderly individuals can convert dietary precursors to UA due to age‐related changes in the microbiome, resulting in limited UA production.^[^ ^51^ ^]^ A recent study identified *Bifidobacterium pseudocatenulatum* INIA P815, a commensal bacterium, as responsible for UA production from ellagic acid.^[^ ^52^ ^]^ This bacterium has been reported to enhance the efficacy of anti‐PDL1 therapy in a CD8^+^ T cell‐dependent manner.^[^ ^7^ ^]^ Our study introduces a different mechanism for Bifidobacterium‐augmented antitumor therapy, raising the possibility of using *Bifidobacterium pseudocatenulatum* INIA P815 as a probiotic to enhance CD8^+^ T cell‐mediated antitumor immunotherapy.

## 4 Conclusion

In summary, our study establishes that UA enhances the persistence and antitumor activity of CD8^+^ T cells by engaging the ERK1/2‐ULK1 cascade. This finding underscores the potential of UA in tumor immunotherapy. Nevertheless, additional research is warranted to ascertain the optimal dosage required to achieve optimal therapeutic outcomes.

## 5 Experimental Section

### Ethics Approval and Consent to Participate

All human PBMCs used in this study were obtained with the approval of the Ethics Committee of Shandong University School of Basic Medical Sciences (License No. ECSBMSSDU2018‐2‐031). All procedures were carried out in accordance with the Helsinki Declaration and with informed consent from healthy volunteers.

### Mice

OT‐I transgenic mice (CD45.1 or CD45.1/2, C57BL/6J background) expressing an H‐2Kb/OVA_257‐264_‐specific TCR were kindly provided by Prof. CE Rudd (University of Cambridge, UK). C57BL/6J (CD45.2) mice were purchased from the GemPharmatech Co., Ltd (Nanjing, Jiangsu, China). Mice were maintained under specific pathogen‐free conditions at the Laboratory Animal Center of Shandong University (Jinan, Shandong, China) under a regular 12‐hour light/12‐hour dark schedule at a constant room temperature (20° to 24 °C). All mice were used at 8 to 12 weeks of age, unless otherwise noted. All animal experiments were performed in strict accordance with the ethical guidelines and were approved by the Animal Care and Use Committee of Shandong University (License No. ECSBMSSDU2018‐1‐014).

### Cell Line

Human T lymphoblastoid Jurkat cells, human Burkitt lymphoma Namalwa, and Raji cells (ATCC, Manassas, Virginia, USA), mouse lymphoma EL4 cells and ovalbumin (OVA)‐expressing EG7 cells were cultured in RPMI 1640 supplemented with 10% (v/v) fetal bovine serum (FBS) and 50 U ml^−1^ penicillin‐streptomycin. OVA‐expressing mouse melanoma B16F10 (i.e., B16‐MO5) cells and human embryonic kidney 293T (HEK293T) cells were cultured in Dulbecco's modified Eagle's medium (DMEM) supplemented with 10% (v/v) FBS and 50 U ml^−1^ penicillin‐streptomycin. All cell lines were authenticated using STR profiling by Tsingke Biotechnology Co., Ltd. (Beijing, China) and routinely tested for mycoplasma infection.

### Reagents

The metabolites were purchased from Sigma‐Aldrich (St. Louis, MO, USA) (Table S1, Supporting Information). AHR inhibitor CH‐223191 (cat#S7711), ERK inhibitor PD0325901 (cat#S1036), and ERK inhibitor SCH772984 (cat#S7101) were purchased from Selleck (Shanghai, China). Autophagy inhibitor Chloroquine (CQ) (cat#HY‐17589A) and 3‐Methyladenine (3MA) (cat#HY‐19312) were purchased from Med Chem Express (Shanghai, China). ULK1 inhibitor MRT68921 (cat#T9142) was purchased from TargetMol (Shanghai, China). The custom‐synthesized peptide Tat‐ERK_156‐166_ (KKRRQRRRYIHSANVLHR) was produced by CUSABIO (Wuhan, Hubei, China).

### NFAT Luciferase Assay

Jurkat‐NFAT‐Luc cells were constructed by lentiviral transfection of pLV‐NFAT‐RE‐Luci containing a nuclear factor of activated T cells (NFAT) response element‐driven luciferase reporter, as previously described.^[^ ^53^ ^]^ Jurat‐NFAT‐Luc cells were stimulated with plate‐bound anti‐CD3 (1 µg ml^−1^) and anti‐CD28 (3 µg ml^−1^) antibodies for 6 h in the presence of 15 microbial metabolites (10 µm) involved in antitumor effects or immune regulation (Table S1, Supporting Information).^[^ ^54^, ^55^ ^]^ Luciferase activity was measured using the Firefly Luciferase Reporter Gene Assay Kit according to the manufacturer's instructions (cat#RG005, Beyotime Inc., Shanghai, China).

### OT‐Ι CTLs’ Generation and Functional Assay

OVA_257‐264_ (SIINFEKL) peptide‐specific OT‐I CD8^+^ CTLs were generated as described previously.^[^ ^56^, ^57^ ^]^ Briefly, splenocytes from OT‐Ι mice were stimulated with 10 nm OVA_257‐264_ peptide in RPMI 1640 medium containing 10% FBS, 50 U ml^−1^ penicillin‐streptomycin, 50 µm 2‐Mercaptoethanol, and 100 U ml^−1^ human recombinant IL‐2 for 3 days, followed by 3 days of culture in the presence of 10 µm UA (Sigma‐Aldrich) to get mature CTLs. In specific experiments, CTLs were cultured with 10 µm CQ, 3‐MA, MRT68921, PD0325901, SCH772984, and CH‐223191. For cytokine secretion assays, CD8^+^ CTLs were restimulated with anti‐CD3 (1 mg ml^−1^) and anti‐CD28 (3 mg ml^−1^) for 4 h in the presence of 10 mg ml^−1^ Brefeldin A (Biolegend, San Diego, CA, *USA*), followed by flow cytometric analysis. For in vitro cytotoxicity assays, luciferase‐expressing B16F10 cells or EL4 cells were pulsed with 10 nm OVA_257‐264_ peptide for 1 h and cocultured with OT‐I CTLs at different cell ratios in RPMI 1640 medium containing 2% FBS at 37 °C for 5 h. Target cells incubated without effector cells were used to measure maximal death RLU (Relative luciferase activity). The percentage of killing was calculated with the following equation:

(1) %specificlysis=[1−(testRLU/maximaldeathRLU)]×100%

### Adoptive Transfer Therapy of Experimental Tumor Model

For subcutaneous tumor models, 1 × 10^6^ EG7 cells or 2 × 10^5^ B16‐MO5 melanoma cells were subcutaneously inoculated into wild‐type C57BL/6J mice on day 0. OT‐I CD8^+^ CTLs were injected intravenously into tumor‐bearing mice on day 10 when the tumor was palpable. Tumor growth was monitored every two or three days using a digital caliper and presented as tumor volume (calculated as width^2^×length/2). Mice with a tumor size > 20 mm along the longest axis will be euthanized by CO_2_ inhalation for ethical consideration. For lung metastasis models, 2 × 10^5^ B16‐MO5 melanoma cells expressing firefly luciferase (B15‐MO5‐Fluc) were intravenously injected into wild‐type C57BL/6J mice on day 0. OT‐I CD8^+^ CTLs (4 × 10^6^) were injected intravenously into tumor‐bearing mice on day 10. Lung metastases were monitored by bioluminescence imaging with the IVIS Spectrum system (PerkinElmer). Luciferase activity levels are expressed as photons emitted per second per centimeter square per steradian, denoted as p/sec/cm^2^/sr. For co‐transfer assay, UA‐treated CTLs (CD45.1/2) were mixed at a 1:1 ratio with control CTLs (CD45.1), followed by adoptive transfer into the same host (tumor‐free or tumor‐bearing C57BL/6 mice, CD45.2).

### Preparation of Human CAR T Cells

Human 19BBz CAR T cells targeting human CD19 were prepared as previously described.^[^ ^23^ ^]^ HEK293T cells were transfected with lentiviral 19BBz CAR expression plasmid together with psPAX2 (Addgene, #12260) and pMD2.G (Addgene, #12259) using Lentifit (Hanbio, China) transfection reagents. Lentivirus‐containing supernatant was harvested 48 and 72 h after transfection, concentrated by ultracentrifugation, and filtered with a 0.22 µm filter. Primary human T cells from the PBMCs of healthy donors were stimulated with anti‐CD3/CD28 beads at a 1:1 ratio (Miltenyi Biotec, Bergisch Gladbach, Germany) in TexMACS GMP medium (Miltenyi Biotec) supplemented with 10% FBS and 50 IU ml^−1^ IL‐2 for 2 days, followed by transduction with concentrated 19BBz CAR lentivirus containing supernatant through spin transduction for 2 h at 32 °C. Then, transduced cells were passaged daily with a fresh culture medium for another 5 days. Transfection efficiency was determined by GFP expression of the CAR plasmid using flow cytometry.

### Functional Assessment of Human CAR T Cells In Vitro

CAR T cells were treated with 10 µm UA or DMSO for 48 h before the functional study. UA or DMSO‐treated CAR T cells were co‐cultured with Namalwa/Raji target cells for 18 h at indicated Effector: Target (E: T) ratios. Subsequently, supernatants were collected to assess IL‐2, IFN‐γ, and TNF‐α production using ELISA kits (Dakewe). The cytotoxic capacity of CAR T cells was determined by flow cytometry based on the ratio of Dil‐labeled target cells to anti‐human CD19‐APC antibody (cat#561742, BD)‐labeled CAR T cells.

### CAR T Cell Therapy Model

The B‐NDG (NOD‐ Prkdc^scid^ IL2rg^tm^ ^1^) mice (Biocytogen, Beijing, China) were intravenously injected with 5 × 10^5^ firefly luciferase‐expressing Namalwa (Namalwa‐Fluc) cells in 200 µl PBS and received PBS, 3 × 10^6^ DMSO‐treated 19BBz CAR T cells, and 3 × 10^6^ UA‐treated 19BBz CAR T cells respectively on days 5 and 10 randomly. Tumor progression was monitored by bioluminescence imaging with IVIS Spectrum system (PerkinElmer) on days 10 and 16, and survival rate was recorded every day. PBMCs were isolated from recipient mice and stained with indicated antibodies for flow cytometric analysis.

### Intragastric Treatment of Experimental Tumor Model with UA

B16F10 (2 × 10^5^) or MC38 tumor cells were injected subcutaneously in the right flank of six‐week‐old male C57BL/6 mice on day 0. The tumor‐bearing mice were treated with UA (sunflower oil as vehicle control, at the indicated dose daily) through *intragastric* administration for 7 days starting from day 10 or 13. Tumor growth was monitored every other day using a digital caliper and presented as tumor volume (calculated as width^2^×length/2).

### Isolation and Analysis of Tumor‐Infiltrating Lymphocytes (TILs)

Subcutaneous tumors were dissected, minced with scissors, and digested in RPMI 1640 medium containing collagenase type II (Sigma, St.Louis, USA), hyaluronidase (Sigma), and DNase I (Sigma) for 1 h at 37 °C, followed by filtration with a 70 µm strainer. Cells were collected by centrifuge at 1000 rpm for 10 min, then resuspended in 40% Percoll (GE Healthcare, Uppsala, Sweden) and carefully layered on 70% Percoll for density centrifugation. TILs were collected from the interface of gradient used for subsequent experiments.

### Flow Cytometric Analysis

All antibodies used in this study for flow cytometry are shown in Table S2 (Supporting Information). For surface staining, cells were labeled with surface markers in the dark for 30 min at 4 °C. For intracellular staining, cells were fixed using IC fixation buffer (cat#00‐8222‐49, eBioscience, Thermo Fisher Scientific Inc., Waltham, MA, USA), permeabilized with Permeabilization Buffer (cat#00‐8333‐56, eBioscience), and stained with indicated antibodies. For transcription factor staining, cells were fixed and permeabilized with Fixation/Permeabilization Concentrate and Diluent buffer (cat#00‐5521, eBioscience). Samples were run on a CytoFLEX S Flow Cytometer (Beckman Coulter Inc., Brea, CA, USA) and analyzed using the CytExpert program (Beckman Coulter) and FlowJo software (version 10).

### Quantitative Polymerase Chain Reaction (q‐PCR) Assay

OVA_257‐264_ (SIINFEKL) peptide‐specific OT‐I CD8^+^ CTLs were stimulated with 10 nm OVA_257‐264_ peptide in RPMI 1640 medium containing 10% FBS, 50 U ml^−1^ penicillin‐streptomycin, 50 µm 2‐Mercaptoethanol, 100 U ml^−1^ human recombinant IL‐2, and 10 µm Urolithin A (UA) (Sigma‐Aldrich) for 72 h. CAR T cells were treated with 10 µm UA or DMSO for 48 h. Total RNA was extracted from CD8^+^ CTLs and CAR T cells that were treated by Mock or UA using TRIzol reagent according to the manufacturer's protocol (cat#15596026, Invitrogen, Thermo Fisher Scientific Inc.). Extracted RNA was quantified and reverse transcribed into cDNA with random primers using the Revert Aid First Strand cDNA Synthesis Kit (cat#K1642, Thermo Fisher Scientific Inc.) according to the manufacturer's manuals. The qPCR was performed with a SYBR Premix Ex Taq II kit (TaKaRa, Japan) using Bio‐Rad CFX Connect (Bio‐Rad Laboratories, Inc., Berkeley, CA, USA) with the following cycle conditions: an initial incubation at 95 °C/3 min and 39 cycles of 95, 60, and 72 °C/20 s. Expression values were normalized to β‐actin, and fold induction was normalized to the control using the 2^−ΔΔCt^ Method. The primers are listed in Table S2 (Supporting Information).

### RNA‐Seq Analysis

CD8^+^ CTLs were treated with DMSO or UA for 72 h. Total RNA was extracted from DMSO or UA‐treated CD8^+^ CTLs with TRIzol (Invitrogen). Total RNAs (2 µg) were used to prepare a stranded RNA sequencing library using a Stranded mRNA Library Prep Kit from DR08502 (Bioyigene) according to the instructions. The library products corresponding to 200–500 bp were enriched, quantified, and sequenced on an Illumina HiSeq 2500 or BGISEQ‐500 by BGI (Shenzhen, Guangdong, China). The gene expression profiles of the mouse T cells exposed to the various treatments were analyzed by RNA sequencing (BGI, Shenzhen, Guangdong, China), and further analysis was performed using the related software Dr. Tom (BGI). In brief, raw sequencing data were first filtered by FastQC; low‐quality reads were discarded, and adaptor sequences were trimmed. After quality filtering, each sample had≈40.4 to 54.8 million clean reads. Clean reads from each sample were mapped to the Mus musculus GRCm38 reference genome using the TopHat (with Bowtie2). Significantly differentially expressed transcripts were screened using the criteria of |fold change (FC)| ≥ 1.5 and *P*‐value ≤ 0.05. Gene set enrichment analysis (GSEA) was performed to identify significantly enriched pathways in the KEGG databases using the Dr. Tom web tool. GO enrichment analysis of differentially expressed genes was curated and visualized using the BGI Interactive analysis platform with a *P*adj (FDR) < 0.05 to determine statistically significant enrichment (https://biosys.bgi.com).

### Synthesis of Magnetite Microspheres Modified with UA

UA (0.002 mmol), 1 mg Fe_3_O_4_‐COOH, 5 mg EDC, and 5 mg HOBt were added into 5 ml DMSO, oscillating away from light at 37 °C for 12 h. Collected via magnetic adsorption, the UA‐modified magnetite microspheres were placed in a specific solution (methanol: water = 1:1) to dialyze for 48 h. After dialysis, the magnetite microspheres were collected using magnetic adsorption and washed three times with deionized water. Finally, the UA‐modified magnetite microspheres were dispersed in deionized water for subsequent use.

### Mass Spectrometry Detection

Mass spectrometry was performed at the National Protein Science Facility, School of Life Science, Tsinghua University. The immunoprecipitated products generated from UA magnetic beads or Fe_3_O_4_ control beads were separated by SDS‐PAGE and Coomassie blue staining. The target protein bands were cut, digested, redissolved in 0.1% trifluoroacetic acid, and loaded onto a mass spectrometer (Orbitrap Fusion, Thermo) for mass spectrum analysis. The MS data were searched against the target protein database from UniProt using an in‐house Proteome Discoverer (Version PD1.4, Thermo Fisher Scientific, USA).

### In Vitro Binding Assay

Commercial purified Flag‐ERK1, Flag‐ERK2, and MEK1 proteins were incubated overnight with Fe_3_O_4_ control beads or UA‐beads at 4 °C. Proteins were boiled with SDS‐loading buffer, subjected to SDS‐PAGE and confirmed by Coomassie Blue staining. Binding proteins were detected by Western blot.

### In Vitro Kinase Assay

For in vitro kinase assays, 1 µg substrates were incubated with 0.5 µg activated kinases in kinase buffer (50 mm Tris‐Cl pH 7.5, 0.1 mm EGTA, 1 mm DTT, 7.5 mm Mg (CH_3_COO)_2_, 2 µm ATP) for 45 min at 30 °C. The kinase reaction was stopped after adding the loading buffer and boiling it for 5 min at 95 °C. After immunoblotting, phosphorylation was detected using an anti‐phosphoserine antibody (Abcam; ab9332) or phospho‐AMPKβ2 (Ser39) (Cell Signaling Technology; cat#82791) Antibody, active ULK1 (1‐649) (Abcam; cat#ab125656), recombinant ULK1 protein (OriGene Technologies; cat#TP315643), active ERK1 (Abcam; cat#ab116536), active MEK1 (Abcam; cat#ab63209), and recombinant AMPKβ2 protein (OriGene Technologies; cat#TP760111).

### Western Blot and Immunoprecipitation

Cells were lysed in RIPA lysis buffer (Beyotime, Shanghai, China) in the presence of 1% protease inhibitor cocktail and 1% phosphatase inhibitors (Sigma‐Aldrich). Samples were separated using SDS‐PAGE, transferred onto polyvinyl difluoride (PVDF) membrane, and blocked with 5% BSA at room temperature for 2 h. Membranes were then incubated with indicated primary antibodies, followed by incubation with horseradish peroxidase (HRP)‐conjugated secondary antibodies (Proteintech). For immunoprecipitation, Cell lysates were incubated with various antibodies and protein G‐coupled sepharose beads (Sigma) or Urolithin A magnetite microspheres, respectively, followed by immunoblotting with indicated antibodies. All antibodies for western blot are listed in Table S2 (Supporting Information).

### Immunofluorescence Staining and Imaging

OT‐I CTLs were treated with DMSO or UA for 16 h followed by fixation for 15 min with 4% paraformaldehyde (PFA) (15 710, Electron Microscopy Science) at room temperature. Cells were permeabilized with permeabilization buffer (Triton X‐100, P0096, Beyotime) for 5 min at room temperature and then blocked with 30% Normal Goat Serum (SL038, Solarbio) for 30 min at room temperature. Cells were stained at 4 °C overnight with the following primary antibodies: anti‐LC3I/II (1:200, PM036, MBL), anti‐ERK1/2 (1:200, ab184699, Abcam), anti‐p‐ERK (1:200, 4377s, Cell Signaling Technology), anti‐ULK1 (1:100, sc‐390904, Santa), anti‐Atg16 (1:200, PM040, MBL). The samples were washed twice with TBST and incubated for 1 h at room temperature with the following secondary antibodies: anti‐rabbit Alexa Fluor plus 488 (1:2,00, SA00006‐2, Proteintech), anti‐rabbit Alexa Fluor plus 594 (1:200, SA00013‐4, Proteintech), anti‐mouse Alexa Fluor plus 488 (1:200, SA00006‐1, Proteintech), anti‐mouse Alexa Flour 594 (1:200, SA00013‐3, Proteintech), and DAPI (C1002, Beyotime). Images were acquired with Fast Airyscan LSM900 Confocal microscope (Zeiss) with 60× oil objective and analyzed using Zen2010 software.

### Seahorse Analysis

Murine CTLs were cultured with or without Urolithin A (10 µm) for 6 days. OCR (in pmol min^−1^) and ECAR (in mpH min^−1^) were measured using the Seahorse XF‐96 metabolic extracellular flux analysis with two different experimental settings: a glycolytic rate assay and a mitochondrial stress test. For the mitochondrial stress test, cells (2 × 105/well) were adhered to Seahorse 96‐well plates pre‐coated with poly‐L‐lysine (Sigma‐Aldrich) in serum‐free XF Base Media (Agilent) containing 25 mm Glucose, 1 mm pyruvate and 2 mm glutamine for 1 h, after which the following compounds were added sequentially: 2.5 µm oligomycin, 2 µm fluoro‐carbonyl cyanide phenylhydrazone (FCCP), and 0.5 µm rotenone plus 0.5 µm antimycin A. The glycolytic rate assay (ECAR) was measured under basal conditions containing 25 mm Glucose for 1 h, after which the following compounds were added sequentially: 10 mm glucose, 0.5 µm rotenone plus 0.5 µm antimycin A, and 50 mm 2‐deoxy‐glucose (2‐DG).

### Statistical Analysis

All statistical analysis was performed using GraphPad Prism 9.0.0 (GraphPad Software, Bethesda, MD, USA). All results were expressed as the mean ± standard error of the mean (SEM). Statistical significance was determined using unpaired two‐tailed Student's *t*‐test or two‐way ANOVA. The survival study was analyzed with the Mantel‐Cox log‐rank test for statistical differences. *P* values < 0.05 were considered statistically significant.

## Conflict of Interest

The authors declare no conflict of interest.

## Author Contributions

S.M., Q.W., and W.W. are co‐first authors and contributed equally to this work. C.M., C.L., and G.W. performed conceptualized and supervised the study. C.M., C.L., G.W., L.G., X.L., and W.W. acquired funding. C.L., S.M., W.Q, and W.W. designed the experiments. S.M., Q.W., W.W., Y.T., J.Z., C.C., X.S., F.Z., L.D., T.W., L.Z., Y.X., and Y.W. performed experiments. W.W., K.Z., H.W., G.W., C.L., and C.M. provided resources. C.L., S.M., Q.W., and W.W. conducted formal data analysis and visualization. X.Y., Z.W., J.W., X.L., L.G., and H.W. provided helpful suggestions in interpreting data and writing. C.L. and S.M. wrote the original draft with the help of Q.W. and W.W. C.L. and C.M. reviewed and edited the manuscript. All authors approved the final version of the manuscript.

## Supporting information

Supporting Information

Supplemental Data 1

## Data Availability

The data that support the findings of this study are available from the corresponding author upon reasonable request.
