# Supplementary material for: Urolithin A Hijacks ERK1/2‐ULK1 Cascade to Improve CD8+ T Cell Fitness for Antitumor Immunity
Source: Adv Sci (Weinh). 2024 Mar 6;11(18):2310065. doi: 10.1002/advs.202310065 (PMC11095213; doi:10.1002/advs.202310065)
Supplement: Supplementary file 1 — Supporting Information [file ADVS-11-2310065-s002.pdf]

ADVANCED SCIENCE
Open Access

Supporting Information

for *Adv. Sci.*, DOI 10.1002/advs.202310065

Urolithin A Hijacks ERK1/2-ULK1 Cascade to Improve $CD8^+$ T Cell Fitness for Antitumor Immunity

Shuaiya Ma, Qi Wu, Wenxian Wu, Ye Tian, Jie Zhang, Chaojia Chen, Xue Sheng, Fangcheng Zhao, Lu Ding, Taixia Wang, Laixi Zhao, Yuying Xie, Yongxiang Wang, Xuetian Yue, Zhuanchang Wu, Jian Wei, Kun Zhang, Xiaohong Liang, Lifen Gao, Hongyan Wang, Guihua Wang, Chunyang Li* and Chunhong Ma**

Supporting Information

Urolithin A hijacks ERK1/2-ULK1 cascade to improve CD8+ T cell fitness for antitumor immunity

Shuaiya Ma[#], Qi Wu[#], Wenxian Wu[#], Ye Tian, Jie Zhang, Chaojia Chen, Xue Sheng, Fangcheng Zhao, Lu Ding, Taixia Wang, Laixi Zhao, Yuying Xie, Yongxiang Wang, Xuetian Yue, Zhuanchang Wu, Jian Wei, Kun Zhang, Xiaohong Liang, Lifen Gao, Hongyan Wang, Guihua Wang[*], Chunyang Li[*], and Chunhong Ma[*]

Corresponding to: Chunhong Ma (machunhong@sdu.edu.cn), Chunyang Li (lichunyang@sdu.edu.cn), and Guihua Wang (ghwang@tjh.tjmu.edu.cn)

Supplementary Methods

Cells Viability assays

100 µl cells were plated into 96-well plates and treated with indicated microbial metabolites for 24 h or 48 h. Cell viability detection was performed with a CellTiter-Lum Plus kit (Beyotime). Luciferase activity was measured using a microplate reader (LB960, Berthold Centro) for ATP chemiluminescence assays.

ELISA

Human CAR T cells were co-cultured with tumor cells (2.5×10^4) in 48-well plates at E: T ratios of 4:1, 2:1 and 1:1 in a total volume of 500 µl without the addition of exogenous cytokines. After incubation for 18 hours, supernatants were collected and used for ELISA. TNF-α, IFN-γ, and IL-2 secretion were detected using ELISA kits (Biolegend, San Diego, CA, USA) according to the manufacturer's instructions. Absorbance was measured using INFINITE 200 PRO.

Mouse p-ERK ELISA assay

Mouse $CD8^+$ cytotoxic T lymphocytes (CTLs) stimulated with anti-CD3/28 antibodies were treated with different UA concentrations for 48 hours. The cells were prepared after sonicating and centrifuged at 4°C. Then, the contents of p-ERK1/2 in supernatants were measured using a Mouse phospho-extracellular signal-regulated kinase (p-ERK)

ELISA Kit (Hengyuan Biotechnology, Shanghai, China). All procedures were conducted according to the manufacturer's instructions.

Cellular Thermal Shift Assay (CESTA assay)

$CD8^+$ CTLs were exposed to 10 μM UA (DMSO as control) for 24 hours. The cells were then collected, counted, evenly distributed, and heated at a specified temperature gradient (42° to 64°C) for 3 minutes to denature the proteins. The treated cells were prepared by flash-freezing in liquid nitrogen and centrifuged at 4°C. Subsequently, ERK1/2 in the supernatants was analyzed by Western blot analysis.

Nuclear and Cytoplasmic Fractionation

Mouse $CD8^+$ CTLs were first treated with or without 10 μM UA for 48 hours. Cell pellets from 2×10^7 cells were washed with PBS buffer and kept at 4°C. Nuclear and cytoplasmic fractions of the identified cells were isolated using a Nuclear and Cytoplasmic Protein Extraction Kit (Boster, Wuhan, China) according to the manufacturer’s instructions.

Flow cytometry-based autophagosome quantification using Cyto-ID

Cyto-ID is a proprietary dye that includes titratable moieties specific for staining autophagic vesicles (Enzo Lifesciences). Briefly, cells were incubated with prepared Cyto-ID Green stain solution for 30 min at 37 °C in the dark with gentle agitation. Cells were then stained with fixable live/dead, stained with surface antibodies, fixed with 1% formaldehyde, and analyzed with a FITC filter (excitation 488 nm, emission 525 nm).

Mitosox staining in $CD8^+$ T cells

Mitochondrial ROS in $CD8^+$ T cells were measured using a mitochondrial superoxide indicator, following the manufacturer's instructions. Briefly, cells were incubated in 5 μM Mito-sox Red at 37 °C for 10 min. Cells were then stained with fixable live/dead,

stained with surface antibodies, fixed with 1% formaldehyde, and followed by washing with PBS three times. Cells were then detected by flow cytometry.

The Assessment of Systemic Toxicity by Body Weight and Hematoxylin and Eosin (H&E) Staining

The systemic toxicity of the UA was also assessed. Briefly, the mice were subcutaneously inoculated with B16F10. Then, the mice were randomly divided into groups. On days 10-13 post-injection, mice were administrated with sunflower oil (Vehicle) or UA (50, 100 and 200 mg/kg) orally every day for 7 days. The body weight of treated mice was monitored. Then, the mice's major organs (heart, liver, spleen, lung, and kidney) were collected, fixed in 4% paraformaldehyde for 24 h, and embedded in paraffin. Paraffin sections (4 μm) were deparaffinized, rehydrated, stained with hematoxylin and eosin (H&E), and imaged using a panoramic scanning and Image Analysis system for High-definition pathological Section (VS120).

Supplementary Figures and Figure Legends

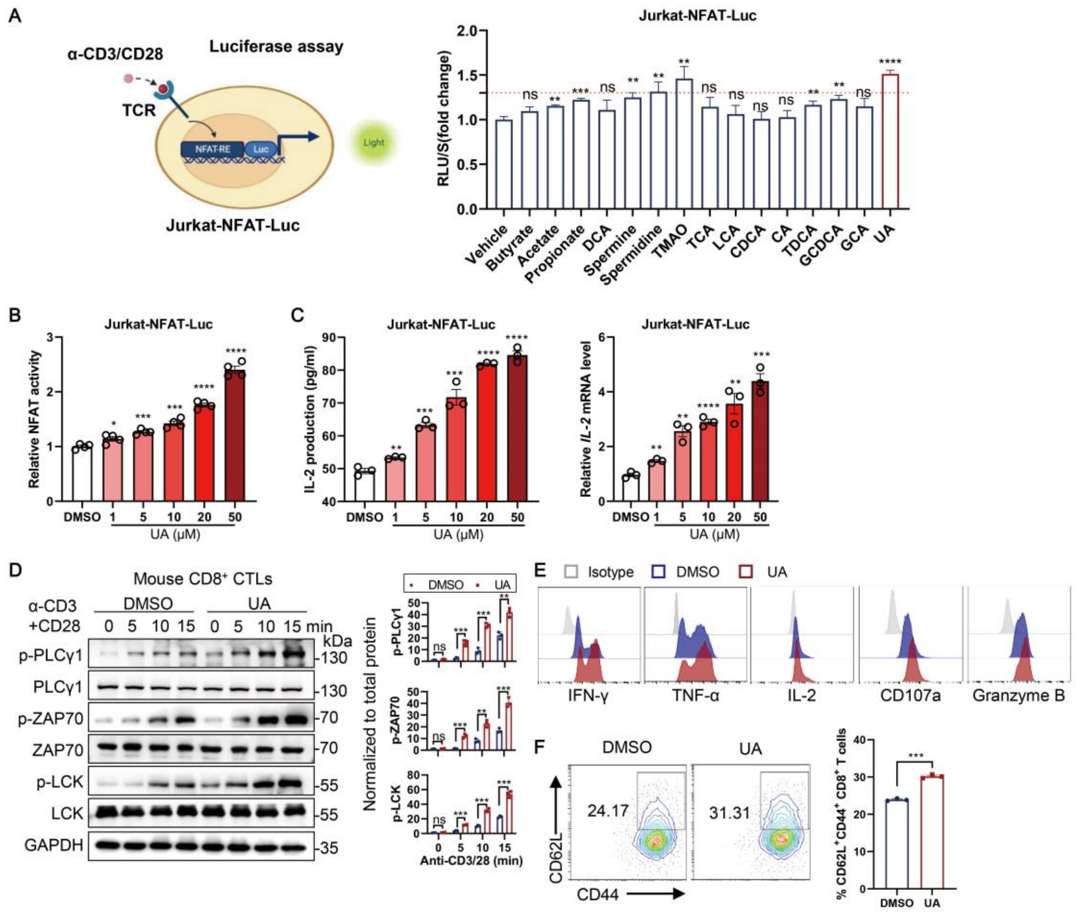

Figure S1. Urolithin A promotes activation of the TCR signaling, related to Figure 1

(**A**) Schematic diagram of NFAT luciferase reporter assay system (left). Jurkat-NFAT-Luc cells were stimulated with 1 μg/ml anti-CD3 and 3 μg/ml anti-CD28 antibodies in the presence of various microbial metabolites at 10 μM for 6 hours, followed by luciferase detection for NFAT activity (right). Data are presented as means ± SEM (*n* = 3) and were analyzed by two-tailed unpaired Student's *t*-test.

(**B and C**) Jurkat-NFAT-Luc cells were pretreated with different doses (1,5,10,20,50 μM) of UA (10 μM) for 2 h, followed by anti-CD3/28 stimulation for 4 hours. NFAT activities were determined by luciferase detection (B). IL-2 production of Jurkat-NFAT-Luc cells in the supernatant was detected using ELISA (C, left). Relative *Il-2*

mRNA expression were detected using qPCR (C, right). Data are presented as means ± SEM (*n* = 3) and were analyzed by two-tailed unpaired Student's *t*-test.

(**D**) Representative immunoblot image (left) and quantification (right; normalized to total protein) of the indicated proteins in DMSO- and 10 μM UA-treated OT-I $CD8^+$ CTLs stimulated with anti-CD3/28 antibodies (1 + 3 μg/ml) for indicated time points. Data are presented as means ± SEM (*n* = 3) and were analyzed by two-tailed unpaired Student's *t*-test. All immunoblots were representative of three independent experiments.

(**E**) Representative flow cytometric histograms of Figure 1E. Flow cytometric analysis of IFN-γ, TNF-α, IL-2, CD107a, and Granzyme B production by DMSO- and UA-treated CTLs stimulated with anti-CD3/28 stimulation for 6 hours.

(**F**) OT-I $CD8^+$ T cells were treated with DMSO or 10 μM UA for 48 hours. The percentage of $CD62L^+CD44^+$ $CD8^+$ T cells was assessed using flow cytometric analysis. Data are presented as means ± SEM (n = 3) and were analyzed by two-tailed unpaired Student's t-test.

All results are representative of at least three independent experiments. * *P* < 0.05, ** *P* < 0.01, *** *P* < 0.001, and **** *P* < 0.0001; ns, no statistically significant.

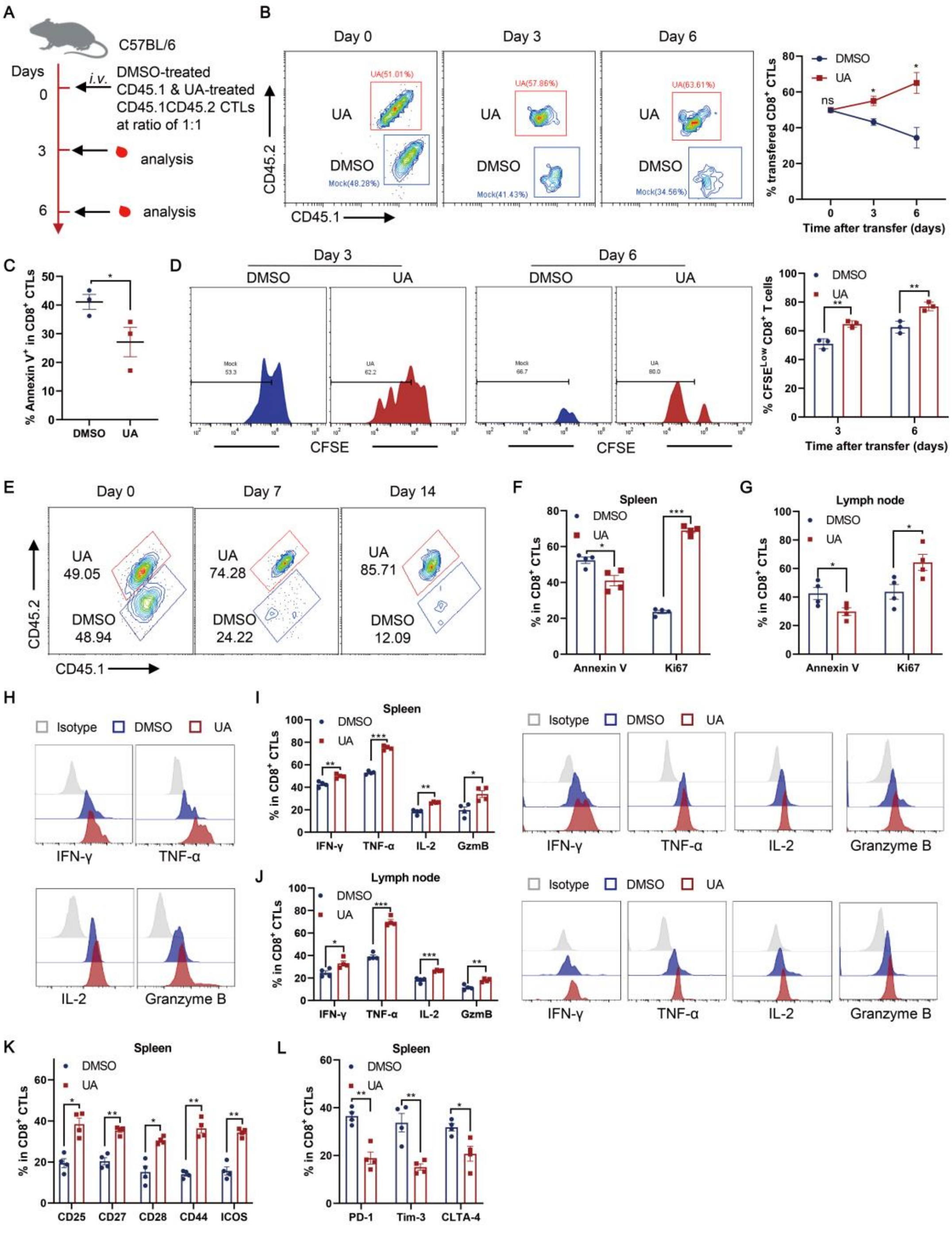

Figure S2. Urolithin A treatment intrinsically promotes the survival and effector function of CD8$^+$ CTLs in *vivo*, related to Figure 1

(**A to D**) Schematic diagram of co-transfer of DMSO- (CD45.1) and 10 μM UA-treated (CD45.1/2) OT-I CTLs at a ratio of 1:1 into CD45.2 C57BL/6 mice (A). Representative contour plots (left) and the proportion of transferred CTLs in peripheral blood were monitored on days 3 and 6 (right) (*n* = 3) (B). Apoptosis of transferred CD8$^+$ CTLs was

evaluated using flow cytometric analysis (*n* = 3) (C). Representative flow cytometric histograms (left) and the statistical graph (right) of CFSE dilution in transferred CTLs on days 3 and 6, which were labeled with CFSE before transfer (*n* = 3) (D). Data are presented as means ± SEM and were analyzed by two-tailed unpaired Student's *t*-test (B to D).

(**E to L**) DMSO- (CD45.1) and 10 μM UA-treated (CD45.1/2) OT-I CTLs were co-transferred to subcutaneous B16-MO5 tumor-bearing C57BL/6 mice at ratio of 1:1 on day 10 post inoculation, related to Figure 1M. Representative contour plots of transferred CTLs in tumors on days 7 and 14 (E) (*n* = 4). Percentages of Annexin V^+ and $Ki67^+$ transferred $CD8^+$ CTLs in the spleen (F) and lymph node (G) (*n* = 4). Representative flow cytometric histograms of IFN-γ^+, TNF-α^+, IL-2^+, and Granzyme B^+ (Gzm B) transferred $CD8^+$ CTLs in tumor (H), related to Figure 1Q. Flow cytometric analysis of IFN-γ, TNF-α, IL-2, and Granzyme B (Gzm B) production in transferred $CD8^+$ CTLs in the spleen (I) and lymph node (J) (*n* = 4). Percentages of $CD25^+$, $CD27^+$, $CD28^+$, $CD44^+$, $CD69^+$, and $ICOS^+$ (K); PD-1^+, Tim-3^+, and CTLA-4^+ (L) transferred $CD8^+$ CTLs in the tumor were assessed on day 7 using flow cytometric analysis (*n* = 4). Data are presented as means ± SEM and were analyzed by two-tailed unpaired Student's *t*-test (F, G, I, J, K, and L).

All results are representative of at least three independent experiments. * $P < 0.05$, ** $P < 0.01$, and *** $P < 0.001$; ns, no statistically significant.

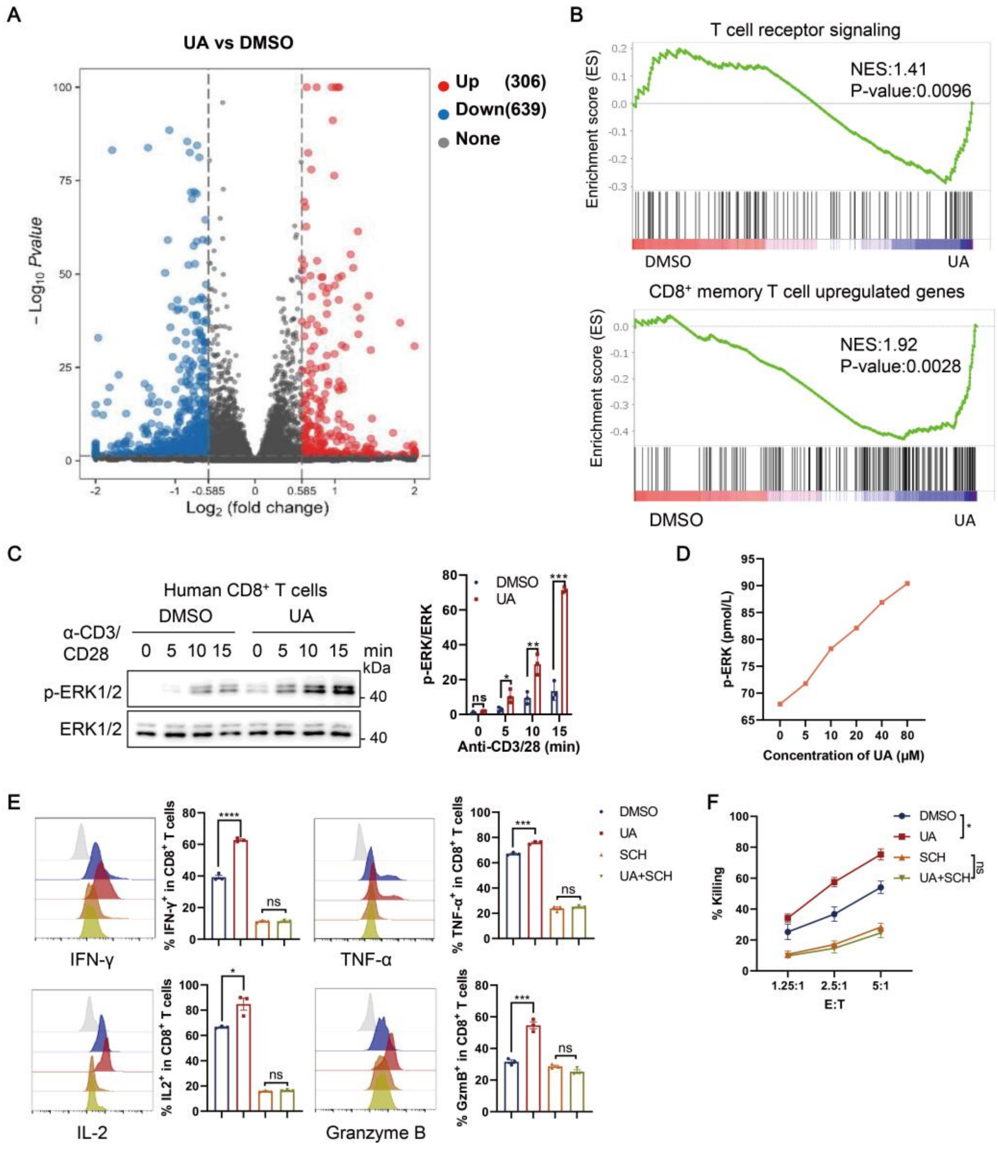

Figure S3. Urolithin A treatment regulates CD8$^+$ T cell function through the ERK pathway, related to Figure 2

(**A**) Volcano plots indicate the significance of upregulated and downregulated genes in DMSO- and UA (10 μM)-treated OT-I CD8$^+$ T cells, with the number of differentially expressed genes. Significance was determined using thresholds of adjusted *P* value < 0.01 and $|\log_2(\text{fold change})| > 0.585$.

(**B**) Gene set enrichment analysis plots of differentially expressed genes in T cell receptor signaling and CD8$^+$ memory T cell pathway between DMSO- and UA-treated

CTLs. RNA-seq data are from one experiment with three technical replicates per sample.

(**C**) Human CD8$^+$ T cells were treated with DMSO and 10 μM UA for 48 hours and stimulated with anti-CD3/CD28 (1 + 3μg/ml) antibodies for indicated time points, followed by immunoblots analysis. Representative immunoblot image (left) and quantification (right, normalized to total ERK1/2) of p-ERK1/2 in DMSO- and UA-treated CD8$^+$ T cells. Quantification data from three independent experiments are presented as means ± SEM (n = 3) and were analyzed by two-tailed unpaired Student's *t*-test.

(**D**) OT-I CD8$^+$ CTLs were treated with different UA concentrations for 48 hours, followed by detecting p-ERK1/2 in supernatants using ELISA.

(**E**) OT-I CD8$^+$ T cells were treated with DMSO or UA (10 μM) in the presence or absence of SCH772984 (SCH, 10μM) for 48 hours, followed by anti-CD3/28 stimulation for 6 hours. Expression of IFN-γ, TNF-α, IL-2, and Granzyme B (Gzm B) in CD8$^+$ T cells was assessed using flow cytometric analysis. Data are presented as means ± SEM (n = 3) and were analyzed by two-tailed unpaired Student's *t*-test.

(**F**) OT-I CD8$^+$ CTLs were treated with DMSO or UA (10 μM) in the presence or absence of SCH772984 (SCH, 10μM) for 48 hours. Subsequently, cytotoxicity of the treated CD8$^+$ CTLs against 10 nM $OVA_{257-264}$ peptide-pulsed EL4 targets at indicated E:T ratios was determined in vitro. Data are presented as means ± SEM (n = 3) and were analyzed by two-way ANOVA.

All results are representative of at least three independent experiments. * $P < 0.05$, ** $P < 0.01$, and *** $P < 0.001$; ns, no statistically significant.

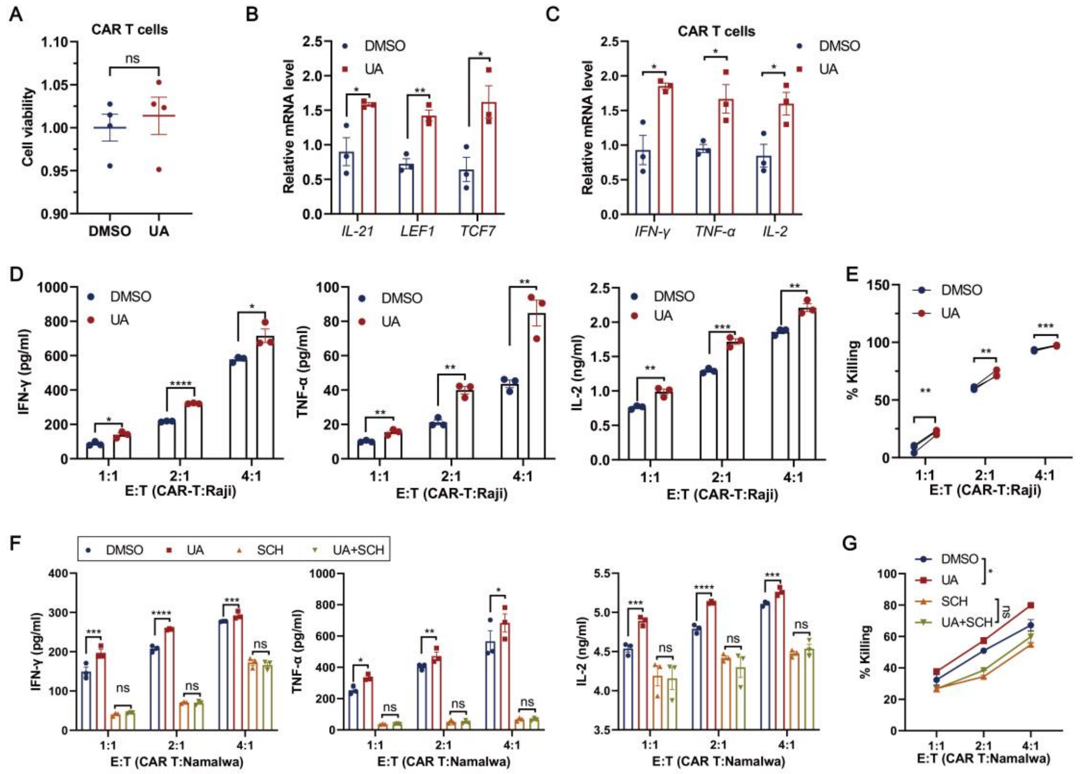

Figure S4. Urolithin A treatment enhances CAR T cell function through ERK, related to Figure 3

(**A**) 19BBz CAR-T cells were treated with DMSO or UA (10 μM) for 48 hours, followed by the detection of cell viability. Data are presented as means ± SEM (*n* = 3) and were analyzed by two-tailed unpaired Student's *t*-test.

(**B and C**) 19BBz CAR-T cells were treated with DMSO or UA (10 μM) for 48 hours and were stimulated with Namalwa cells for 24 h. Relative mRNA expression of *IL-21*, *LEF1* and *TCF7* (B); *IFN-γ*, *TNF-α*, and *IL-2* (C) were detected using qPCR. Data are presented as means ± SEM (*n* = 3) and were analyzed by two-tailed unpaired Student's *t*-test.

(**D and E**) 19BBz CAR-T cells were treated with DMSO or UA (10 μM) for 48 hours, followed by stimulated with Raji cells at E:T ratios of 1:1, 2:1 and 4:1 for 18 hours. Cytokine productions (IFN-γ, TNF-α, and IL-2) in the supernatants were detected using ELISA (D). Cytotoxicity of CAR T cells against Raji cells in vitro was assessed using

flow cytometry (E). Data are presented as means ± SEM (*n* = 3) and were analyzed by two-tailed unpaired Student's *t*-test.

(**F and G**) 19BBz CAR-T cells were treated with DMSO or UA (10 μM) in the presence or absence of ERK inhibitor SCH772984 (SCH, 10μM) for 48 hours, followed by stimulated with Namalwa cells at E:T ratios of 1:1, 2:1 and 4:1 for 18 hours. Cytokine production (IFN-γ, TNF-α, and IL-2) of 19BBz CAR T cells in the supernatant was detected using ELISA (F). Cytotoxicity of 19BBz CAR T cells against Namalwa cells was determined in vitro using flow cytometry (G). Data are presented as means ± SEM (*n* = 3) and were analyzed by two-tailed unpaired Student's *t*-test (F) and two-way ANOVA (G).

All results are representative of at least three independent experiments. * $P < 0.05$, ** $P < 0.01$, *** $P < 0.001$, and **** $P < 0.0001$; ns, no statistically significant.

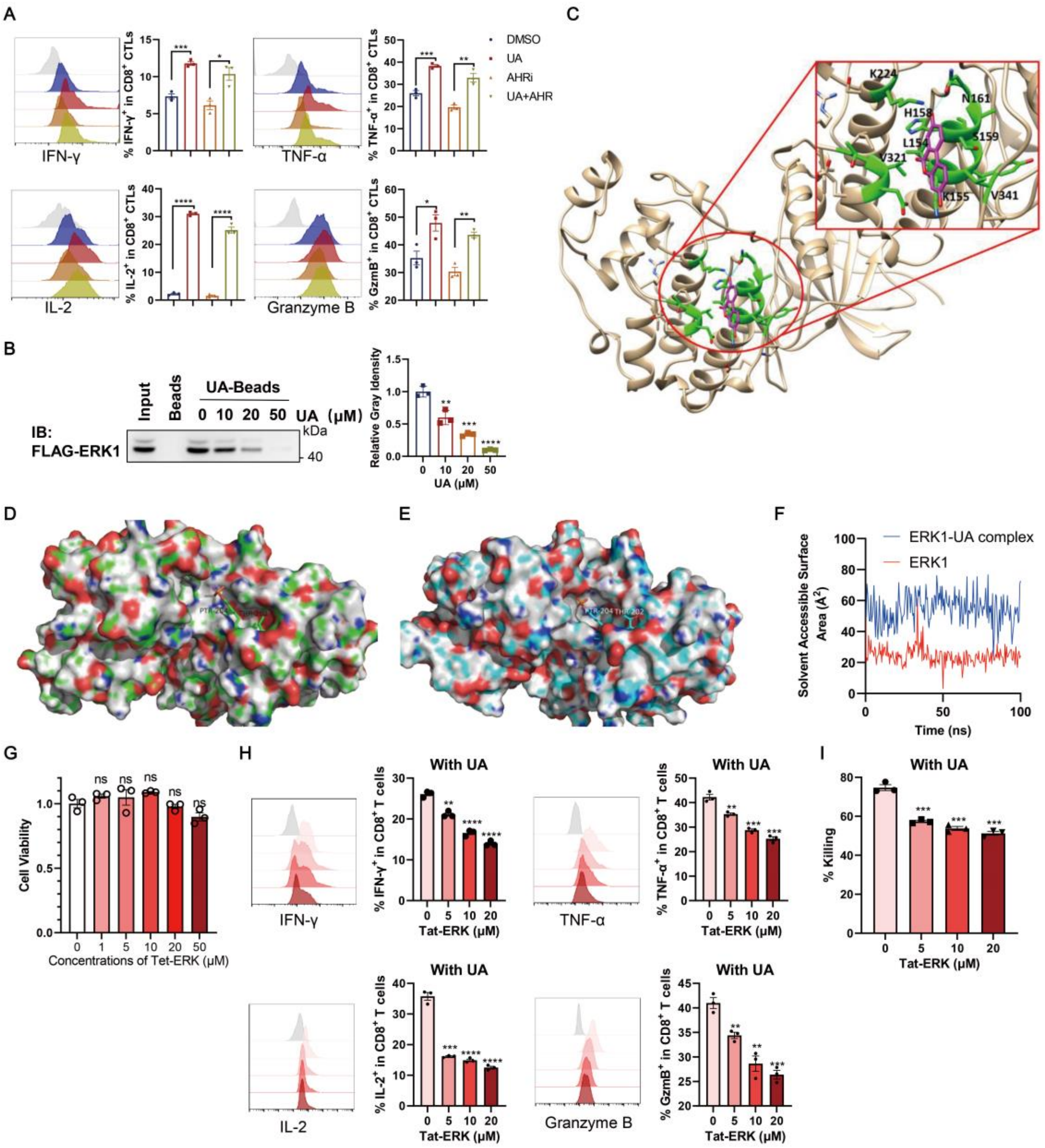

Figure S5. Urolithin A binds ERK1/2 to facilitate their activation, related to Figure 4

(**A**) OT-I CD8$^+$ T cells were treated with DMSO or UA (10 μM) in the presence or absence of AHR inhibitor CH-223191 (AHRi, 10μM) for 48 hours, followed by anti-CD3/28 stimulation for 6 hours. Expression of IFN-γ, TNF-α, IL-2, and Granzyme B (Gzm B) in CD8$^+$ T cells was assessed using flow cytometric analysis. Data are presented as means ± SEM (n = 3) and were analyzed by two-tailed unpaired Student's t-test.

(**B**) The Flag-tagged ERK1 was transfected into HEK293T cells, and the whole cell

lysate was collected for a pull-down assay using control beads or UA-beads in the presence of different doses (10, 20, and 50 μM) of UA, followed by immunoblot analysis. Quantification data from three independent experiments are presented as means ± SEM (n = 3) and were analyzed by two-tailed unpaired Student's t-test.

(**C**) Interactions of UA and ERK1 by docking analysis. Schematic representation of UA binding to ERK1 was shown.

(**D to F**) The molecular dynamics (MD) simulations of UA-ERK1 interaction. Representative conformations of the apo ERK (D) and ERK bound with UA (E). The protein structure is shown on the surface, with red representing O atoms, blue representing N atoms, white representing H atoms, and C atoms shown in green in D and cyan in E. The side chains of residue Thr 202 and PTR 204 are demonstrated in sticks. Comparing D and E, the side chain of Thr 202 in C is buried deeper by the surrounding residues, while its counterpart in E is more exposed to the solvent. Solvent accessible surface area (SASA) plot of ERK1 and complex with UA (F).

(**G**) Viability of OT-I CTLs treated with different doses of Tat-ERK (1, 5, 10, 20, and 50 μM) peptide for 48 hours. Data are presented as means ± SEM (n = 3) and were analyzed by two-tailed unpaired Student's t-test.

(**H**) OT-I CTLs were treated with UA (10 μM) in the presence of different doses (5, 10, and 20 μM) of Tat-ERK peptide *in vitro* for 48 hours and then stimulated with anti-CD3/CD28 antibodies for 6 hours. Flow cytometric analysis assessed the production of IFN-γ, TNF-α, IL-2, and granzyme B in CD8+ CTLs. Data are presented as means ± SEM (n = 3) and were analyzed by two-tailed unpaired Student's t-test.

(**I**) OT-I CTLs were treated with UA (10 μM) in the presence of different doses (5, 10, and 20 μM) of Tat-ERK peptide *in vitro* for 48 hours. Cytotoxicity of the treated CTLs against 10 nM $OVA_{257-264}$ peptide-pulsed EL4 targets was determined in vitro. Data are presented as means ± SEM (n = 3) and were analyzed by two-tailed unpaired Student's t-test.

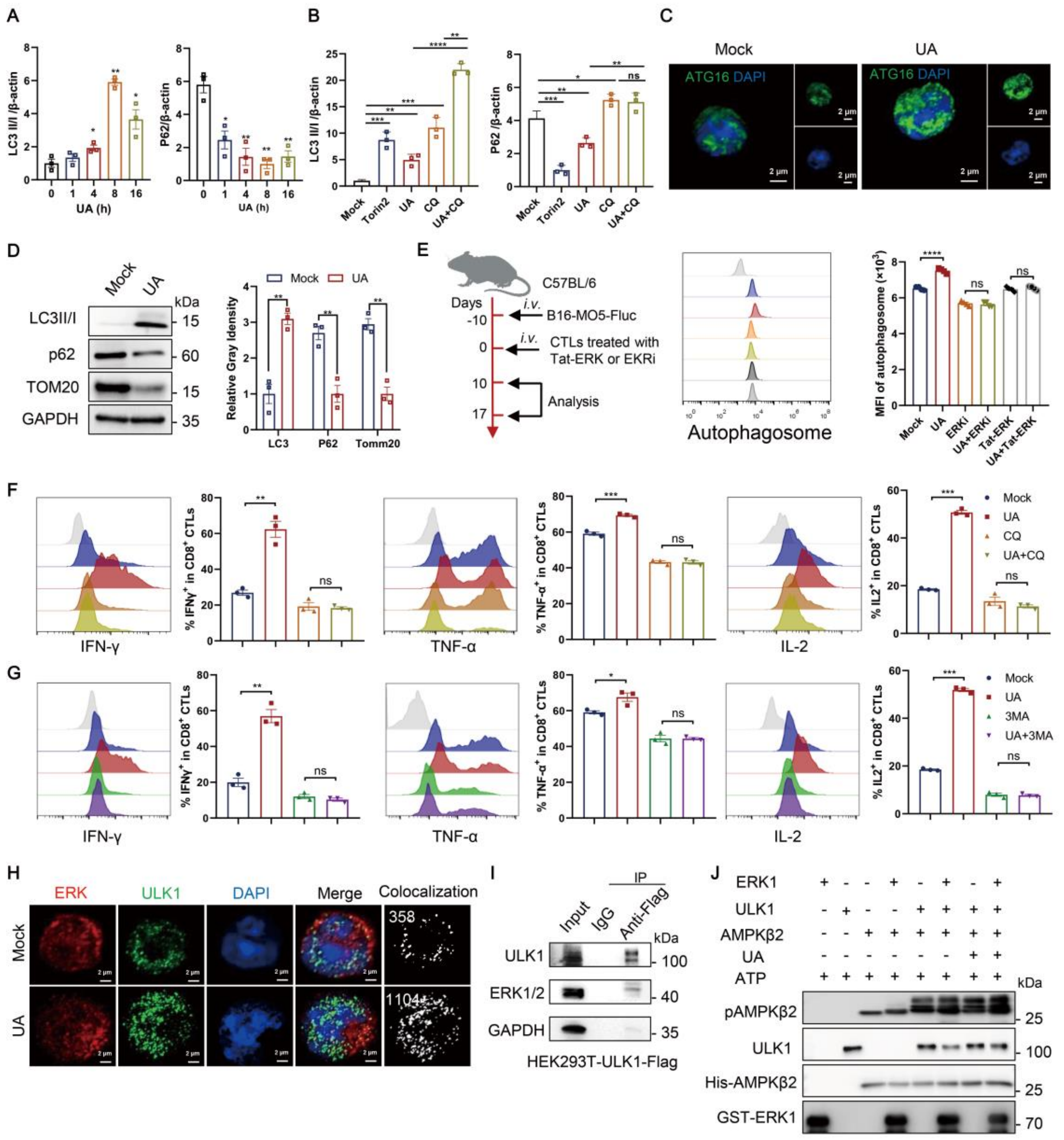

Figure S6. Urolithin A-primed ERK1/2 triggers ULK1 activation and downstream autophagy flux to promote CD8^{+} T cell function, related to Figure 5

(**A**) Quantification data of Figure 5A from three independent experiments are presented as means ± SEM (n = 3) and were analyzed by two-tailed unpaired Student's t-test.

(B) Quantification data of Figure 5B from three independent experiments are presented as means ± SEM (n = 3) and were analyzed by two-tailed unpaired Student's t-test.

(**C**) Representative images of ATG16 staining in OT-I CD8^{+} T cells treated with 10 μM UA or DMSO for 48 hours. The immunofluorescence images show ATG16 (green) and DAPI (blue) in CTLs. White scale bars, 2 μm. All immunofluorescence experiments

were performed three times, independently, with similar results.

(**D**) OT-I $CD8^+$ CTLs were treated with 10 μM UA or DMSO for 48 hours, followed by immunoblot analysis. Quantification data of indicated protein (normalized to GAPDH) from three independent experiments are presented as means ± SEM (n = 3) and were analyzed by two-tailed unpaired Student's t-test.

(**E**) OT-I $CD8^+$ CTLs were treated with 10 μM UA or DMSO in the presence or absence of PD0325901 (ERKi, 10 μM) or Tat-ERK (10 μM) for 48 hours and transferred to B16-MO5-Fluc lung metastases-bearing C57BL/6 mice. Autophagosomes in transferred $CD8^+$ CTLs were assessed using flow cytometry. Data are presented as means ± SEM (n = 4) and were analyzed by two-tailed unpaired Student's t-test.

(**F**) OT-I $CD8^+$ CTLs were treated with 10 μM UA or DMSO for 48 hours in the presence or absence of chloroquine (CQ, 10 μM) for the last 4 hours and then stimulated with anti-CD3/28 for 6 hours. Production of IFN-γ, TNF-α, IL-2, and granzyme B (Gzm B) in $CD8^+$ CTLs was assessed using flow cytometric analysis. Data are presented as means ± SEM (n = 3) and were analyzed by two-tailed unpaired Student's t-test. This experiment was repeated three times independently.

(**G**) OT-I $CD8^+$ CTLs were treated with 10 μM UA or DMSO for 48 hours in the presence or absence of 3-MA (0.5 mM) for the last 4 hours and then stimulated with anti-CD3/28 for 6 hours. Production of IFN-γ, TNF-α, IL-2, and granzyme B (Gzm B) in $CD8^+$ CTLs was assessed using flow cytometric analysis. Data are presented as means ± SEM (n = 3) and were analyzed by two-tailed unpaired Student's t-test. This experiment was repeated three times independently.

(**H**) OT-I CTLs were treated with 10 μM UA or DMSO for 48 hours, followed by anti-CD3/28 stimulation for 10 min. The co-localization of phosphorylated ERK1/2 (p-ERK1/2) and ULK1 was assessed by immunofluorescence staining analysis. Immunofluorescence images show p-ERK (red), ULK1 (green), and DAPI (blue) in CTLs. White scale bars, 2μm. This experiment was repeated three times independently.

(**I**) Co-immunoprecipitation analysis of Flag-tagged ULK1 (Flag-ULK1) and

endogenous ERK1/2 in HEK293T cells transduced with Flag-ULK1 using anti-Flag antibodies. All immunoblots were performed three times, independently, with similar results. This experiment was repeated three times independently.

(**J**) Representative immunoblot images of Figure 5L. This experiment was repeated three times independently.

All results are representative of at least three independent experiments. * $P < 0.05$, ** $P < 0.01$, *** $P < 0.001$ and ****$P < 0.0001$; ns, no statistically significant.

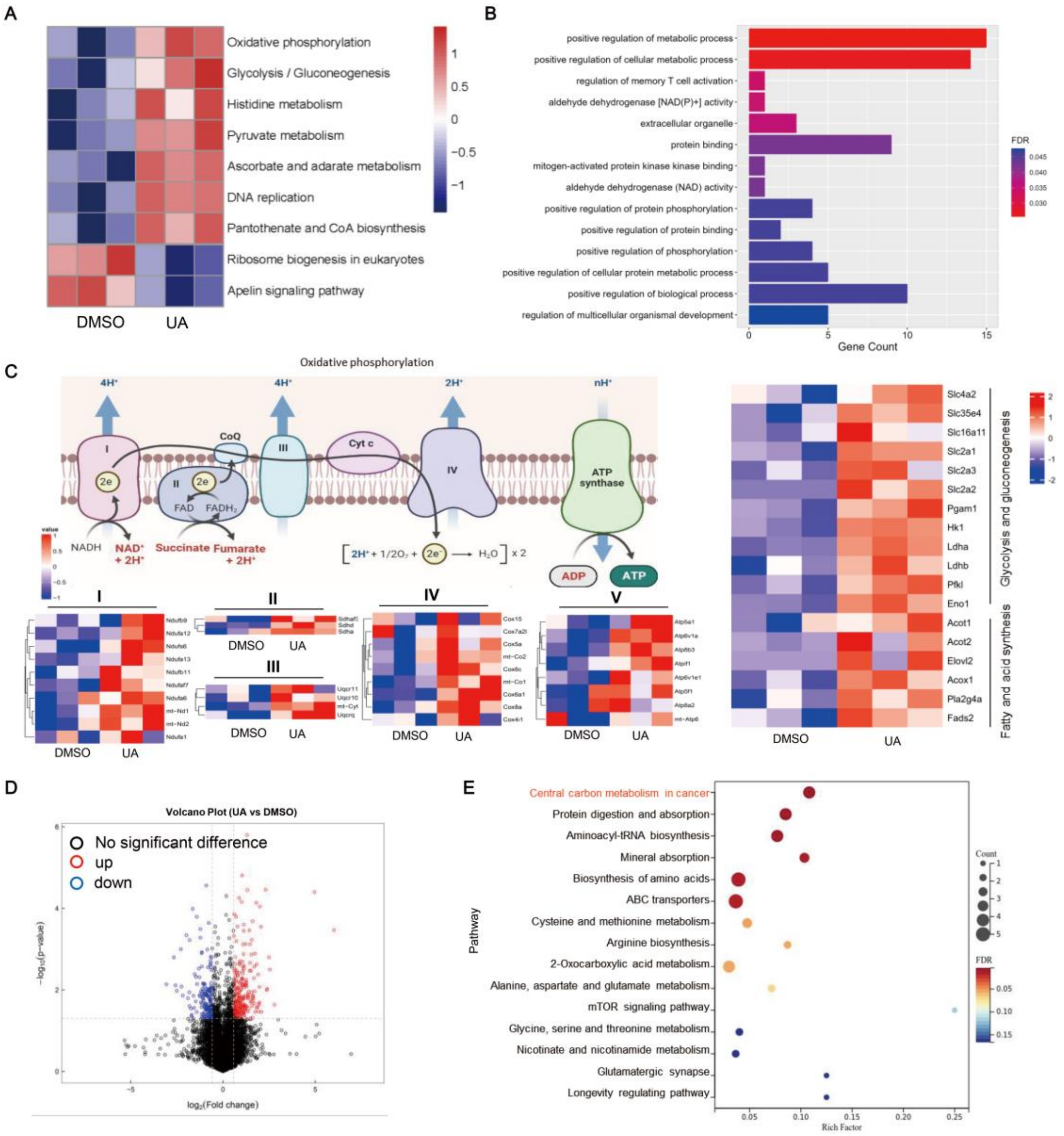

Figure S7. UA-ERK1/2-ULK1 cascade-mediated autophagy regulates cellular metabolism, related to Figure 6

(**A to C**) OT-I CD8$^+$ CTLs were treated with DMSO and UA (10μM) for 48 hours, followed by RNA-seq analysis. The data are from one experiment with three technical replicates per sample. GSVA analysis of RNA-seq data of DMSO- and UA-treated OT-I CD8$^+$ CTLs (A). Gene Ontology (GO) Biological Process enrichment results in the differentially expressed genes (DEGs) with RNA-seq data of DMSO- and UA-treated OT-I CD8$^+$ CTLs. Heat map illustrating the average transcript expression of the indicated genes in different metabolic pathways (C). Rows represent averaged z-scores.

Asterisks denote transcripts with significant differential expression by DESeq2 (FDR < 0.05).

(**D**) Volcano plots of the metabolic intermediates between DMSO- and UA-treated $CD8^+$ CTLs. The data are from one experiment with three technical replicates per sample.

(**E**) KEGG pathway enrichment analysis of the significantly altered metabolite pathways identified with the comparison between DMSO- and UA-treated $CD8^+$ CTLs. The data are from one experiment with three technical replicates per sample.

All results are representative of at least three independent experiments.

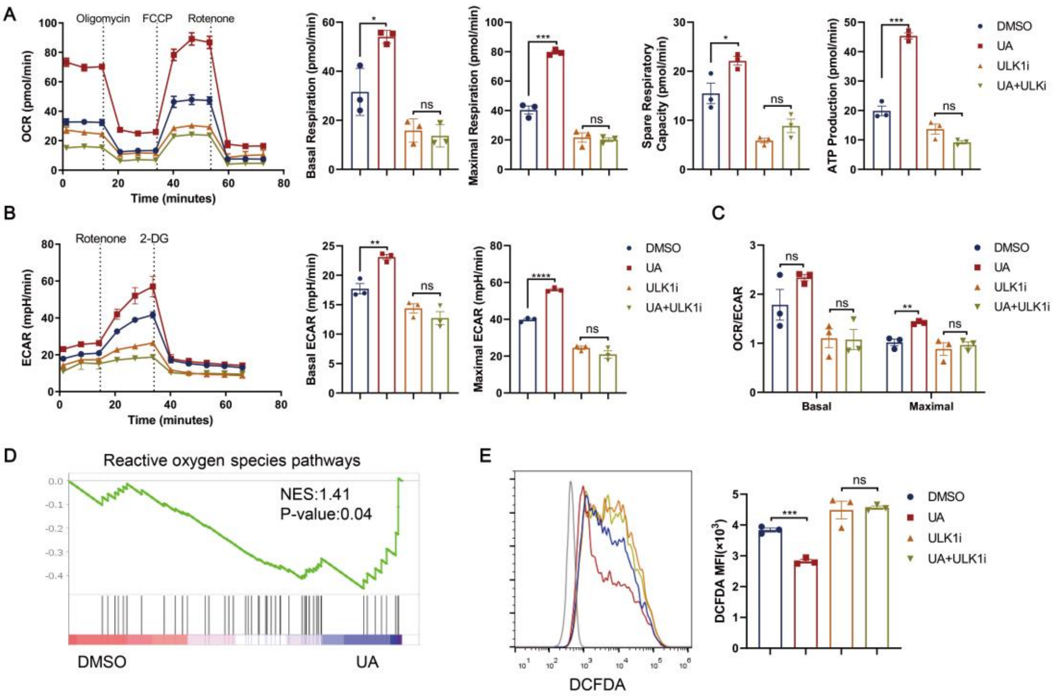

Figure S8. UA-ERK1/2-ULK1 cascade-mediated autophagy regulates cellular metabolism and ROS levels, related to Figure 6

(**A**) OT-I CD8$^+$ CTLs cells were treated with 10 μM UA or DMSO for 48 hours in the presence or absence of ULK1 inhibitor MRT68921 (ULK1i, 5 μM) for the last 4 hours. Oxygen consumption rate (OCR) was measured in treated CD8+ CTLs under basal and stimulated conditions with oligomycin, FCCP, and rotenone. Basal respiration, maximal respiration, spare respiratory capacity statistics, and ATP production were shown. Data are presented as means ± SEM (n = 3) and were analyzed by two-tailed unpaired Student's t-test.

(**B**) OT-I CD8$^+$ CTLs cells were treated with 10 μM UA or DMSO for 48 hours in the presence or absence of ULK1 inhibitor MRT68921 (ULK1i, 5 μM) for the last 4 hours. Extracellular acidification rate (ECAR) of the treated OT-I CTLs was measured under basal and stimulated conditions with glucose, rotenone, and 2-deoxy-glucose (2-DG). Basal and maximal ECAR were shown. Data are presented as means ± SEM (n = 3) and were analyzed by two-tailed unpaired Student's t-test.

(**C**) Ratios of OCR to ECAR in DMSO- and UA-treated CD8$^+$ CTLs were shown. Data are presented as means ± SEM (*n* = 3) and were analyzed by two-tailed unpaired Student's *t*-test.

(**D**) GSEA plot of reactive oxygen species pathway with DEGs between DMSO- and UA-treated OT-I CTLs. The data are from one experiment with three technical replicates per sample.

(**E**) OT-I CD8$^+$ CTLs were treated with 10 μM UA or DMSO for 48 hours, followed by ULK1i (MRT68921, 5 μM) treatment for 4 hours. ROS (DCFDA) were assessed using flow cytometry. Data are presented as means ± SEM (*n* = 3) and were analyzed by two-tailed unpaired Student's *t*-test.

All results are representative of at least three independent experiments. * $P < 0.05$, ** $P < 0.01$, and *** $P < 0.001$; ns, no statistically significant.

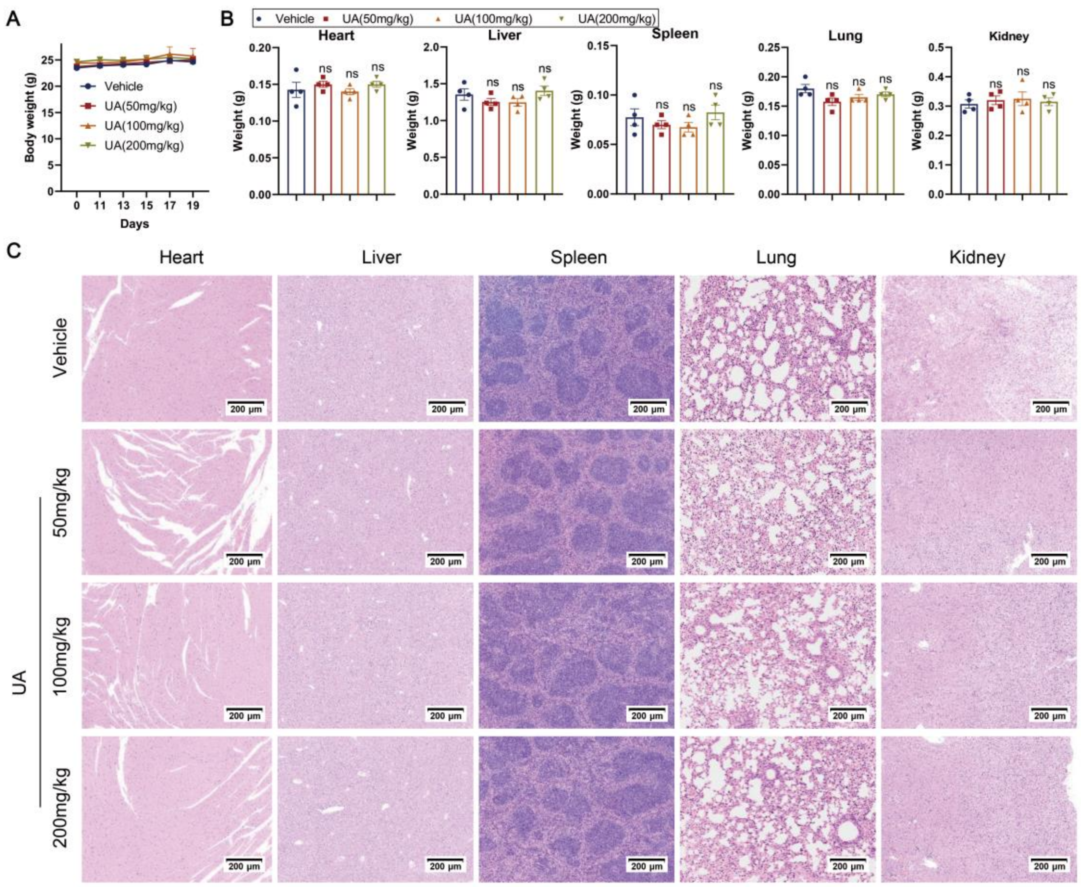

Figure S9. No significant tissue damage was observed in mice after the oral administration of Urolithin A, related to Figure 7A

C57BL/6 mice were inoculated subcutaneously with 2 × 10^5 B16F10 melanoma cells on day 0 and treated with daily intragastric administration of UA at different doses (50, 100, and 200 mg/kg, sunflower oil as vehicle control) from day 12 to 19 (*n* = 4 mice per group).

(**A**) The body weight of the indicated groups was monitored. Data are presented as means ± SEM (*n* = 4 mice per group) and analyzed by the Two-way ANOVA.

(**B**) The heart, liver, spleen, lung, and kidney weights of the indicated groups were measured after mice sacrifice. Data are presented as means ± SEM (*n* = 4 mice per group) were analyzed by two-tailed unpaired Student's *t*-test

(**C**) Representative H&E staining images of indicated organs were shown (Black bar = 50 μm).

All results are representative of at least three independent experiments. * $P < 0.05$, ** $P < 0.01$, and *** $P < 0.001$; ns, no statistically significant.

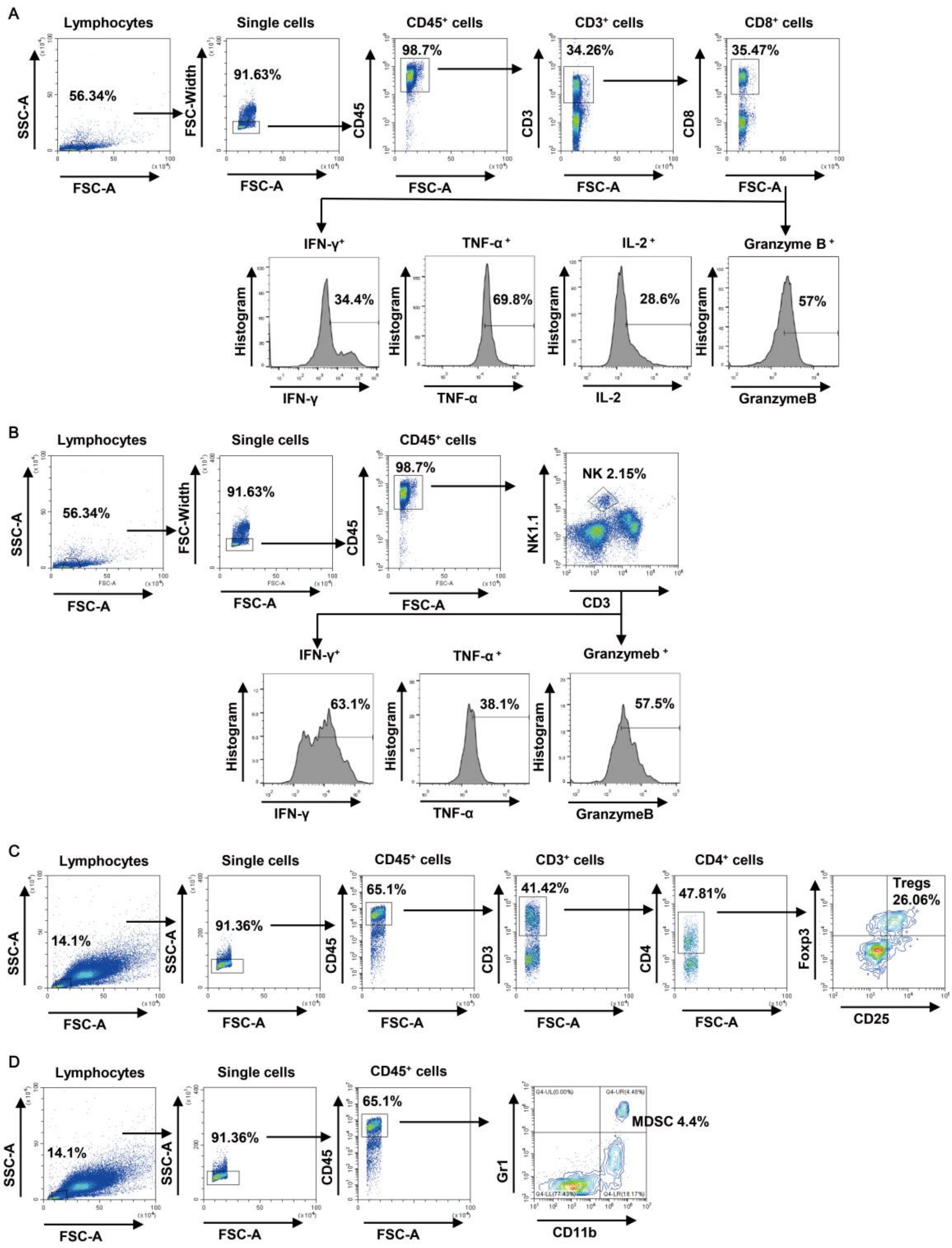

Figure S10. Gating strategy of flow cytometric analysis, related to Figure 7D, E; Figure S11

Gating strategy for cytokine production by CD8$^+$ T cells (A) and NK cells (B); CD4$^+$ T cells and Tregs (C); MDSCs (D)

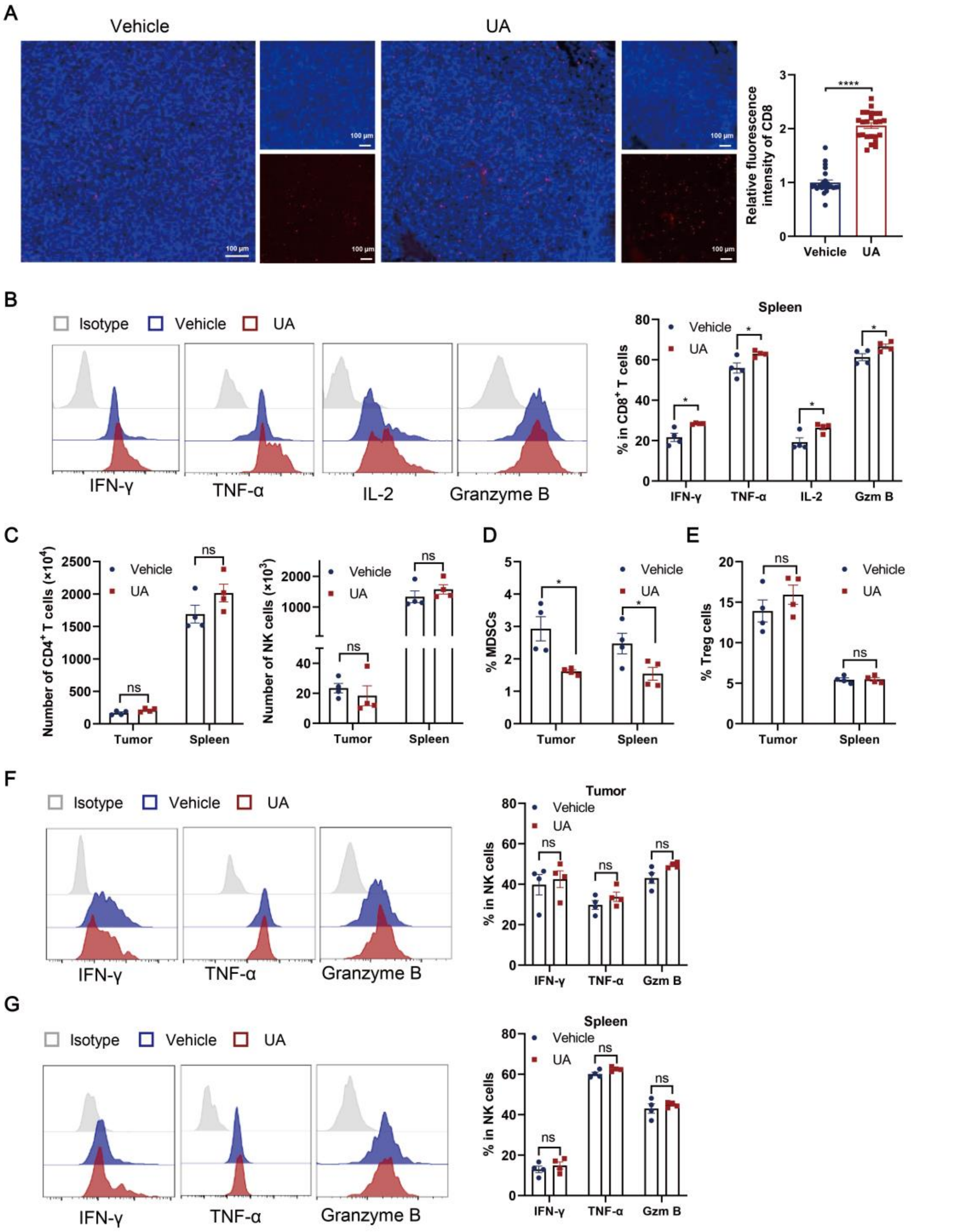

Figure S11. Oral administration of Urolithin A promotes antitumor immune responses, related to Figure 7D to H

Subcutaneous B16F10 tumor models were treated with daily intragastric administration of UA at 50 mg/kg (sunflower oil as vehicle control) from day 12 to 19.

(**A**) Representative immunofluorescence images of CD8 staining with tumor. Images show CD8 (red) and DAPI (blue). White scale bars, 100 μm. Quantification of red CD8

fluorescence intensity normalized to control was shown. Data are presented as means ± SEM (*n* = 24 images per group) and were analyzed by two-tailed unpaired Student's *t*-test.

(**B**) Percentages of IFN-γ^+, TNF-α^+, IL-2^+, and Granzyme B^+ $CD8^+$ T cells in the spleen were estimated by flow cytometric intracellular staining (*n* = 4 mice per group). A two-tailed unpaired Student's *t*-test was used for statistical analysis. Data are presented as means ± SEM and were analyzed by two-tailed unpaired Student's *t*-test.

(**C**) The number of $CD4^+$ T cells and NK cells in the tumor and spleen were analyzed using flow cytometry (*n* = 4 mice per group). Data are presented as means ± SEM and were analyzed by two-tailed unpaired Student's *t*-test.

(**D and E**) Percentages of MDSCs (D) and Treg (E) in the tumor and spleen were analyzed using flow cytometry (*n* = 4 mice per group). Data are presented as means ± SEM and were analyzed by two-tailed unpaired Student's *t*-test.

(**F and G**) IFN-γ, TNF-α, and Granzyme B production by NK cells in the tumor (F) and spleen (G) (*n* = 4 mice per group). Data are presented as means ± SEM and were analyzed by two-tailed unpaired Student's *t*-test.

All results are representative of at least three independent experiments. * $P < 0.05$, ** $P < 0.01$, and *** $P < 0.001$; ns, no statistically significant.

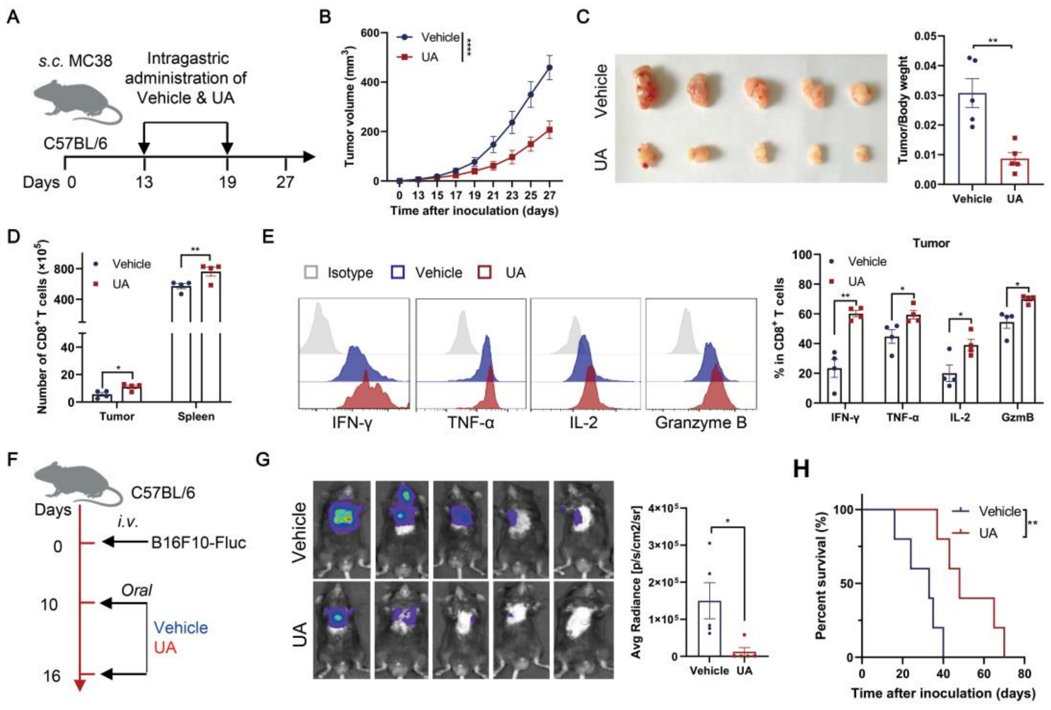

Figure S12. Urolithin A exhibits antitumor activity in MC38 tumor mice, related to Figure 7

(A to E) C57BL/6 mice were subcutaneously inoculated with 2 × 10^5 MC38 cells on day 0 and treated with daily intragastric administration of UA at 50 mg/kg from day 13 to 19 (A). Tumor volumes were monitored (*n* = 5 mice per group) (B). Tumor images and tumor-to-body weight ratios were shown (*n* = 5 mice per group) (C). Numbers of $CD8^+$ T cells in the tumor and spleen were analyzed using flow cytometry (*n* = 5 mice per group) (D). Expression of IFN-γ, TNF-α, IL-2, and Granzyme B in tumor-infiltrating $CD8^+$ T cells was assessed using flow cytometry (*n* = 4 mice per group) (E). Data are presented as means ± SEM and were analyzed by two-way ANOVA (B) and two-tailed unpaired Student's *t*-test (C to E).

(**F to H**) C57BL/6 mice bearing B16-MO5-Fluc lung metastases were treated with sunflower oil or UA (F). Tumor growth was assessed by detecting luciferase activity in the lung (G). and survival curve (H) was monitored (*n* = 5 mice per group). Data are presented as means ± SEM and were analyzed by two-tailed unpaired Student's *t*-test (G) and Log-rank test (H).

All results are representative of at least three independent experiments. * $P < 0.05$, ** $P < 0.01$, *** $P < 0.001$, and **** $P < 0.0001$; ns, no statistically significant.

Table S1. A list of metabolites

Metabolite	Abbreviations	CAS No.	Identifier	Company
Butyrate	Nabu	156-54-7	cat#303410	Sigma
Acetate	Acetate	127-09-3	cat#S5636	Sigma
Propionate	Propionate	137-40-6	cat#P5436	Sigma
Deoxycholic acid	DCA	302-95-4	cat#30970	Sigma
Spermine	Spermine	71-44-3	cat#S4264	Sigma
Spermidine	Spermidine	334-50-9	cat#85578	Sigma
Trimethylamine oxide	TMAO	1184-78-7	cat#317594	Sigma
Taurocholic acid	TCA	345909-26-4	cat#T4009	Sigma
Lithocholic acid	LCA	434-13-9	cat#L6250	Sigma
Chenodeoxycholic Acid	CDCA	474-25-9	cat#C9377	Sigma
Cholic acid	CA	81-25-4	cat#C1129	Sigma
Tauroursodeoxycholic acid	TDCA	207737-97-1	cat#T0875	Sigma
Glycochenodeoxycholic acid	GCDCA	16564-43-5	cat#G0759	Sigma
Glycocholic acid	GCA	1192657-83-2	cat#G2878	Sigma
Urolithin A	UA	1143-70-0	cat#SML1791	Sigma

Table S2. Experimental materials

**Regent or Resource**	**Source**	**Identifier**
**Antibodies anti-mouse**		
anti-mouse CD45(percpcy5.5)	Biolegend	cat#103132
anti-mouse CD4(PE/cy7)	eBioscience	cat#25-0041-82
anti-mouse CD8(PE)	eBioscience	cat#100708
anti-mouseCD8(percpcy5.5)	eBioscience	cat#45-0081-82
anti-mouse CD8(APC)	Biolegend	cat#100712
anti-mouse CD8(FITC)	Biolegend	cat#100706
anti-mouse CD45.1(percpcy5.5)	Biolegend	cat#110728
anti-mouse CD45.1(APC-eFluor780)	eBioscience	cat#4336598
anti-mouse CD45.1(PE)	Biolegend	cat#110708
anti-mouse CD45.1(FITC)	Biolegend	cat#110706
anti-mouse CD45.2(FITC)	Biolegend	cat#109806
anti-mouseCD45.2(BV421)	eBioscience	cat#48-0454-82
anti-mouse/human CD44(FITC)	Biolegend	cat#103006
anti-human/mouse CD44(PE)	Biolegend	cat#103008
anti-mouse CD62L(PE/cy7)	Biolegend	cat#104418
anti-human/mouse/rat CD27(percpcy5.5)	Biolegend	cat#124213

anti-mouse CD25(FITC)	eBioscience	cat#53-0253-82
anti-mouseLy6G/Ly6C (Gr1)-(PE)	Biolegend	cat#108408
Anti-mouseCD11b(PE/cy7)	Biolegend	cat#101216
anti-mouse IFN-γ(PE/cy7)	BD Bioscience	cat#557649
anti-mouse IFN-γ(APC)	eBioscience	cat#17-7311-82
anti-mouse IFN-γ(BV421)	eBioscience	cat#11-9668-82
anti-mouse TNF-α(PE)	eBioscience	cat#12-7321-81
anti-mouse IL-2(FITC)	Biolegend	cat# 503806
anti-mouse CD107a(BV421)	Biolegend	cat#121617
anti-mouse perforin (APC)	Biolegend	cat#154304
anti-mouse/human Granzyme B(PE)	Biolegend	cat#372207
anti-mouse Foxp3(eFlour660)	eBioscience	cat#53-0253-82
BV421 anti-human/mouse T-bet	Biolegend	cat#644816
**Antibodies anti-human**		
anti-human CD3(FITC)	Biolegend	cat#300406
anti-human/mouse CD44(APC)	eBioscience	cat#17-0441-81
anti-Human CD62L(PE)	BD Bioscience	cat#560966
anti-human CD8a(BV421)	Biolegend	cat#301036
anti-human CD8a(PE/cy7)	eBioscience	cat#25-0088-42

anti-human TNF-α(APC)	Biolegend	cat#502912
anti-human IFN-γ(BV421)	Biolegend	cat#502532
**Western Blot**		
Anti-GAPDH(1E6D9)	Proteintech	cat#60004-i-ig
Beta Actin Monoclonal antibody	Proteintech	cat#66009-1-Ig
Phospho-PLCγ1(Tyr783) (D6M9S) Rabbit mAb	Cell Signaling Technology	cat#14008
Phospho-Lck(Tyr505) antibody	Cell Signaling Technology	cat#2751
p44/42MAPK(Erk1/2)antibody	Cell Signaling Technology	cat#9102
Phosphor-p44/42MAPK (Erk1/2)	Cell Signaling Technology	cat#9196
Phospho-Zap-70(Tyr319)/Syk (Tyr352) (65E4) Rabbit mAb	Cell Signaling Technology	cat#2701
SQSTM1/P62	Cell Signaling Technology	cat#5114
LC3B Rabbit mAb	ABclonal	cat#A19665
TOM20 Rabbit mAb	ABclonal	cat#A19403
ATG5 Polyclonal Antibody	Proteintech	cat#10181-2-AP

Beclin-1(D40C5) Rabbit mAb	Cell Signaling Technology	cat#3495
ULK1 (D8H5) Rabbit mAb	Cell Signaling Technology	cat#8054
Phospho-ULK1(Ser757) (D7O6U) Rabbit mAb	Cell Signaling Technology	cat#14202
Phospho-AMPKβ2(Ser39) Antibody	Cell Signaling Technology	cat#82791
Phospho-Atg14(Ser29) (D4B8M) Rabbit mAb	Cell Signaling Technology	cat#92340
Phospho-Atg13(Ser355) (E4D3T) Rabbit mAb	Cell Signaling Technology	cat#46329
Phospho-Beclin-1(Ser15) (D4B7R) Rabbit mAb	Cell Signaling Technology	cat#84966
Anti-DDDDK-tag	MBL	cat#M185-3
**Immunofluorescence staining**		
ATG16	MBL	cat#PM040
Anti-LC3 pAb	MBL	cat#PM036
AlexaFluor488Goat anti-Rabbit IgG	Proteintech	cat#SA00006-2
**Primer sequences**	**Forward primer (5'-3')**	**Reverse primer (5'-3')**

m-IFN-γ	TGAGTATTGCCAAG TTTGAGGTCA	CGGCAACAGCTGGT GGAC
m-Granzyme B	CCACTCTCGACCCT ACATGG	GGCCCCCAAAGTGA CATTTATT
m-perforin 1 (Prf1)	AGCACAAGTTCGTG CCAGG	GCGTCTCTCATTAG GGAGTTTTT
m-TNF-α	AGTGACAAGCCTGT AGCCC	GAGGTTGACTTTCT CCTGGTAT
m-IL-2	GGAGCAGCTGTTGA TGGACCTAC	AATCCAGAACATGC CGCAGAG
m-actin	GGCTGTATTCCCCT CCATCG	CCAGTTGGTAACAA TGCCATGT
h- IFN-γ	TGGAGACCATCAAG GAAGAC	GCGTTGGACATTCA AGTCAG
h-TNF-α	AGCCCATGTTGTAG CAAACC	GGAAGACCCCTCCC AGATAG
h-IL-2	AACTCCTGTCTTGC ATTGCAC	GCTCCAGTTGTAGC TGTGTTT
h-actin	AGTTGCGTTACACC CTTTC	CCTTCACCGTTCCA GTTT

**shRNA sequence**		
m-Lv-shATG5-1	5′-<br>CCTTTGGCCTAAGA<br>A GAAA-3′	
m-Lv-shATG5-2	5′-<br>CATCTGAGCTACCC<br>GGATA-3′	
**Others**		
Percoll	GE	17-0891-09
DMSO	Solarbio	D8371
Ficoll	TianjinHaoyang	LTS1077
Collagenase type II	Worthington	LS004176
DNase I	ThermoFisher Scientific	EN0521
